# Safety and cost-effectiveness of individualised screening for diabetic retinopathy: the ISDR open-label, equivalence RCT

**DOI:** 10.1007/s00125-020-05313-2

**Published:** 2020-11-04

**Authors:** Deborah M. Broadbent, Amu Wang, Christopher P. Cheyne, Marilyn James, James Lathe, Irene M. Stratton, John Roberts, Tracy Moitt, Jiten P. Vora, Mark Gabbay, Marta García-Fiñana, Simon P. Harding, the ISDR Study Group

**Affiliations:** 1 Department of Eye and Vision Science, Institute of Life Course and Medical Sciences, University of Liverpool, Member of Liverpool Health Partners, Liverpool, UK; 2 St Paul’s Eye Unit, Liverpool University Hospitals Foundation Trust, Member of Liverpool Health Partners, Liverpool, UK; 3 Department of Biostatistics, University of Liverpool, Member of Liverpool Health Partners, Liverpool, UK; 4 Clinical Trials Research Centre, Liverpool, UK; 5 Division of Rehabilitation, Ageing and Wellbeing, School of Medicine, University of Nottingham, Nottingham, UK; 6 Gloucestershire Retinal Research Group, Cheltenham General Hospital, Cheltenham, UK; 7 Mersey Diabetes Support Group, Liverpool, UK; 8 Department of Diabetes and Endocrinology, Royal Liverpool University Hospital, Liverpool, UK; 9 Department of Health Services Research, University of Liverpool, Member of Liverpool Health Partners, Liverpool, UK; 10 Brownlow Health Centre, Member of Liverpool Health Partners, Liverpool, UK

**Keywords:** Diabetic retinopathy, Individualised, Personalised, Risk-based, Screening, Systematic, Variable interval

## Abstract

**Aims/hypothesis:**

Using variable diabetic retinopathy screening intervals, informed by personal risk levels, offers improved engagement of people with diabetes and reallocation of resources to high-risk groups, while addressing the increasing prevalence of diabetes. However, safety data on extending screening intervals are minimal. The aim of this study was to evaluate the safety and cost-effectiveness of individualised, variable-interval, risk-based population screening compared with usual care, with wide-ranging input from individuals with diabetes.

**Methods:**

This was a two-arm, parallel-assignment, equivalence RCT (minimum 2 year follow-up) in individuals with diabetes aged 12 years or older registered with a single English screening programme. Participants were randomly allocated 1:1 at baseline to individualised screening at 6, 12 or 24 months for those at high, medium and low risk, respectively, as determined at each screening episode by a risk-calculation engine using local demographic, screening and clinical data, or to annual screening (control group). Screening staff and investigators were observer-masked to allocation and interval. Data were collected within the screening programme. The primary outcome was attendance (safety). A secondary safety outcome was the development of sight-threatening diabetic retinopathy. Cost-effectiveness was evaluated within a 2 year time horizon from National Health Service and societal perspectives.

**Results:**

A total of 4534 participants were randomised. After withdrawals, there were 2097 participants in the individualised screening arm and 2224 in the control arm. Attendance rates at first follow-up were equivalent between the two arms (individualised screening 83.6%; control arm 84.7%; difference −1.0 [95% CI −3.2, 1.2]), while sight-threatening diabetic retinopathy detection rates were non-inferior in the individualised screening arm (individualised screening 1.4%, control arm 1.7%; difference −0.3 [95% CI −1.1, 0.5]). Sensitivity analyses confirmed these findings. No important adverse events were observed. Mean differences in complete case quality-adjusted life-years (EuroQol Five-Dimension Questionnaire, Health Utilities Index Mark 3) did not significantly differ from zero; multiple imputation supported the dominance of individualised screening. Incremental cost savings per person with individualised screening were £17.34 (95% CI 17.02, 17.67) from the National Health Service perspective and £23.11 (95% CI 22.73, 23.53) from the societal perspective, representing a 21% reduction in overall programme costs. Overall, 43.2% fewer screening appointments were required in the individualised arm.

**Conclusions/interpretation:**

Stakeholders involved in diabetes care can be reassured by this study, which is the largest ophthalmic RCT in diabetic retinopathy screening to date, that extended and individualised, variable-interval, risk-based screening is feasible and can be safely and cost-effectively introduced in established systematic programmes. Because of the 2 year time horizon of the trial and the long time frame of the disease, robust monitoring of attendance and retinopathy rates should be included in any future implementation.

**Trial registration:**

ISRCTN 87561257

**Funding:**

The study was funded by the UK National Institute for Health Research.

**Figure:**
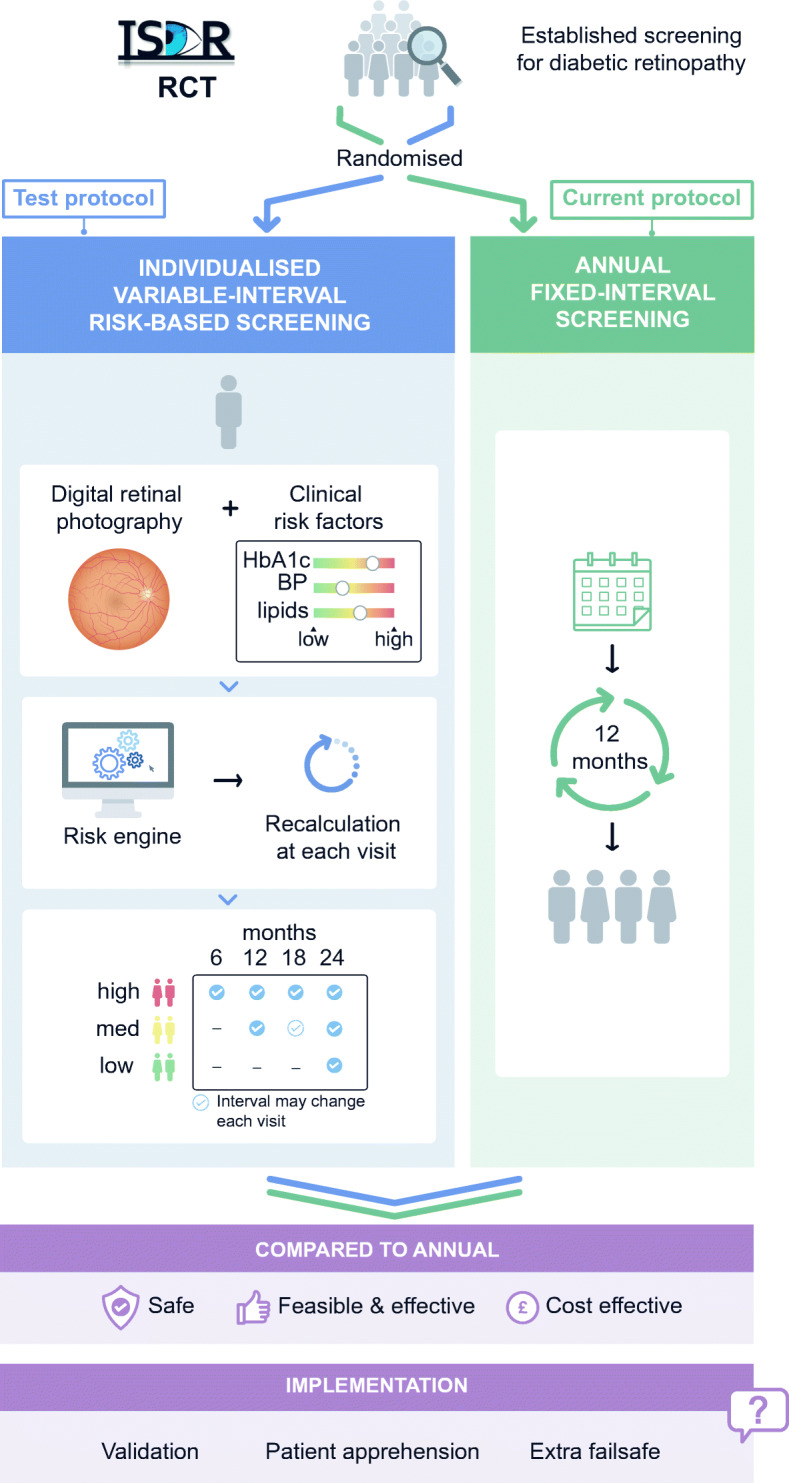
Graphical abstract

**Supplementary Information:**

The online version of this article (10.1007/s00125-020-05313-2) contains peer-reviewed but unedited supplementary material, which is available to authorised users.

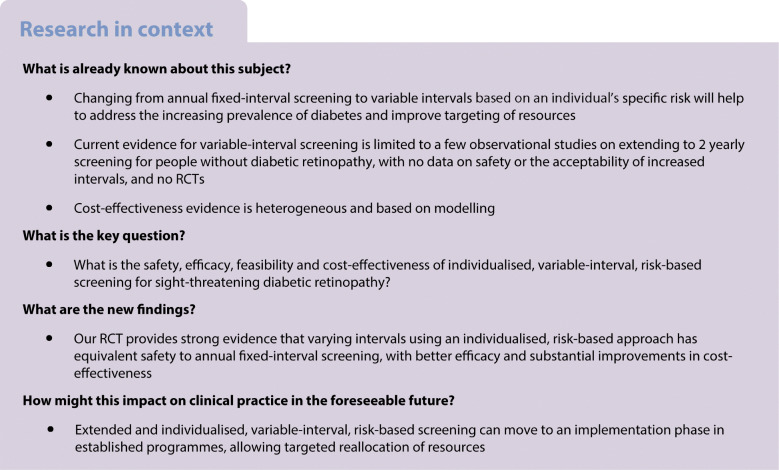



## Introduction

Early detection of sight-threatening diabetic retinopathy (STDR) at a stage allowing timely intervention, through systematic programmes of screening, is universally recognised to be important in preventing visual impairment [1] and reducing its associated costs, but approaches vary greatly worldwide. The frequency of screening has to date been annual, based on consensus, and this remains the recommendation in major guidelines [2, 3]. However, the prevalence of diabetes is increasing rapidly [4], increasing the requirement for screening, and resources are stretched.

Extending the interval between screening episodes offers potential cost savings. Some developed countries have recommended or implemented 2 yearly and sometimes longer intervals for people at low risk of progression. Much evidence supporting extended intervals comes from observational studies from areas with low incidence rates of STDR [5–8] and from modelling studies [9]. Safety concerns have been highlighted by a recent systematic review that called for RCTs and cost-effectiveness evidence [10], and recent failures in cancer screening [11]. In addition, the feasibility of connecting large and disparate datasets is considered challenging [12].

Based on our previous incidence data [5] we designed an RCT (the Individualised Screening for Diabetic Retinopathy [ISDR] study) to investigate the safety, efficacy and cost-effectiveness of extending screening intervals in low-risk individuals with diabetes, with more frequent intervals for those at high risk. We used the emerging methodology and technologies of personalised risk prediction [13, 14] and data linkage to develop an individualised, variable-interval, risk-based screening approach. Individualised clinical care offers opportunities for improved patient engagement. We also wanted to test the feasibility and stability of linking routine data across varying National Health Service (NHS) domains in an integrated approach. We tested the hypothesis of equivalence between attendance rates, as a primary measure of safety, for individualised and annual screening.

## Methods

### Study design and participants

Individuals with diabetes attending for diabetic retinopathy screening were invited to participate in a single-site, two-arm, parallel-assignment, equivalence RCT conducted in all community screening clinics in the Liverpool Diabetic Eye Screening Programme, which is part of the English National Diabetic Eye Screening Programme. The rationale, design and methodology have been published elsewhere [15], and the protocol and statistical and health economics analysis plans are available online [16]. In brief, inclusion criteria comprised: age 12 years or older, attending for retinal screening during the recruitment period, registered with a participating general practitioner, with no retinopathy or retinopathy/maculopathy less than the definition of screen-positive diabetic retinopathy, gradable digital retinal images in both eyes and did not opt out of the data warehouse (see below).

The English National Screening Committee definition of a screen-positive result was used, comprising any of: (1) moderate preproliferative diabetic retinopathy (R2) (equivalent to moderate non-proliferative diabetic retinopathy) or worse (any of: multiple deep blot haemorrhages, venous beading, intraretinal microvascular abnormalities, or worse); (2) new proliferative diabetic retinopathy (R3A); (3) maculopathy (M1) (any of: exudates ≤1 disc diameter (DD) from the foveal centre, group exudates ≥1/2 disc area (DA) ≥1 DD from the foveal centre, haemorrhage ≤1 DD from the foveal centre if visual acuity ≥+0.30 log minimal angle of resolution); (4) ungradable images; or (5) other significant sight-threatening disease [17, 18]. The definition of STDR was met when either retinopathy or maculopathy, as defined above, was confirmed on clinical examination by a retinal specialist (R2, R3A and/or M1 in England).

A patient and public involvement (PPI) group was embedded in all aspects of the study design, delivery and interpretation. The Liverpool Clinical Trials Research Centre developed electronic case report forms, information systems, quality assurance systems and systems to minimise operational bias (see electronic supplementary material [ESM] Methods, Clinical Trials Research Centre procedures). Ethics approval was by Preston NHS Research Ethics Committee (14/NW/0034).

The trial opened on 1 May 2014. Follow-up was for a minimum 24 months plus a 90 day window to attend the screening invitation. Participants were recruited by trained researchers at their screening appointment and all provided written informed consent. For children aged 12–15 years, proxy consent was by the parent/guardian with, where appropriate, assent from the child. Trial management is described in ESM Methods, Trial management.

Participants were allocated 1:1 to annual screening (control arm, current care) or individualised, risk-based, variable-interval screening with recall at 6, 12 or 24 months for those at high, medium and low risk, respectively. A purpose-built, dynamic data warehouse linking primary and secondary care demographic, retinopathy and systemic risk factor data populated the baseline and follow-up electronic case report forms (OpenClinica, v3.12; OpenClinica, USA). Block randomisation generated by an independent statistician was conducted using a bespoke, validated electronic system at the Clinical Trials Research Centre, with stratification by clinic and age using random blocks of four and six for participants aged ≥16 years, and blocks of two for those aged <16 years to account for small numbers. Screening staff and clinical assessors were observer-masked to the intervention arm, risk calculation and screening interval.

### Procedures

Each participant’s risk of becoming screen-positive was assessed by a risk-calculation engine (RCE) that was specifically developed for the RCT and is described in detail elsewhere [19]. Briefly, the RCE uses data on retinopathy levels and demographic and clinical risk factors from the local population and the individual to estimate the likelihood of progression for that individual over a given time period.

An RCE development dataset comprised 5 years’ retinopathy, demographic and clinical data to 4 February 2014 held in the ISDR data warehouse from 11,806 individuals with diabetes. Participants and their general practitioners had agreed to data sharing. The RCE is a Markov multi-state model, with states defined by retinopathy level (both eyes) and transitions dependent on risk factors including historical retinopathy data. Candidate risk factors were identified in collaboration with the PPI group and selected as informative using the corrected Akaike’s information criterion method. Risk factors in the development dataset that were identified and included in the model were age, time since diagnosis of diabetes, HbA_1c_, systolic BP and total cholesterol. The time periods of 6, 12 and 24 months and a risk threshold of 2.5% were selected as agreed with the PPI group. The RCE showed good discriminatory ability. Corrected AUCs for 6, 12 and 24 months were 0.88 (95% CI 0.83, 0.93), 0.90 (95% CI 0.87, 0.93) and 0.91 (95% CI 0.87, 0.94), respectively. Sensitivities and specificities for a 2.5% risk were, respectively, 0.61 and 0.93 for 6 months, 0.67 and 0.90 for 12 months, and 0.82 and 0.81 for 24 months. Using the 2.5% threshold, the corrected C-index for the model was 0.687 [19].

At each screening visit during the trial, the RCE calculated a participant’s risk of becoming screen-positive using automatic exchanges of retinopathy data from the screening software (OptoMize v4.3; EMIS Health, UK) and risk factor data held in the data warehouse and randomisation databases. The data warehouse was updated with clinical data from primary care every 2 months. Participants were allocated to a high-, medium- or low-risk group against the 2.5% threshold. The screening interval could change at each follow-up visit. Participants in the control arm continued with invitations to annual screening, with risk recorded for future analysis.

Participants who were screen-positive attended for slit-lamp biomicroscopy to determine the presence of STDR (true positive). Participants with a false-positive result were reconsented and re-entered the trial. Participants were free to withdraw consent at any time without providing a reason.

### Outcomes

The primary outcome of attendance at first follow-up visit (6, 12 or 24 months) assessed the safety of individualised screening. Non-attendance was defined as failure to attend any appointment within 90 days of the follow-up invitation, irrespective of the number of invitations.

Secondary outcomes measuring safety and efficacy reported here include STDR, visual acuity (recorded as log of the minimum angle of resolution), visual impairment (visual acuity ≥+0.30 and ≥0.50), screen-positive results and rates of retinopathy treatment over the 24 months (see ESM Methods, Secondary outcomes). Quality-adjusted life-years (QALYs) were used to produce cost-effectiveness estimates.

### Statistical analysis

Our primary hypothesis was that attendance rates at first follow-up would be equivalent in the two arms with a 5% equivalence margin. The estimated minimum sample size was 4460 (90% power, 2.5% one-sided type 1 error, assuming the same attendance rate in both arms and allowing for 6% per annum loss over 24 months). Further details, including of a sample size review during the recruitment phase, are in ESM Methods, Sample size. Our secondary hypothesis was that STDR detection was non-inferior in the individualised arm at a prespecified margin of 1.5%.

Primary equivalence and non-inferiority analyses followed a per-protocol approach supported by secondary intention-to-treat analyses [20]. Adherence to protocol for attendance was considered at the first follow-up visit and by 24 months (+90 days) for STDR. Multiple imputations generated using generalised linear models (GLMs) dependent on baseline characteristics (PROC multiple imputation; SAS v9.3; SAS Institute, USA), assessed the effect of missing values on both per-protocol and intention-to-treat datasets.

Within the three risk groups of the individualised arm, equivalence in attendance rates between the two arms and non-inferiority in detection of STDR were explored. Participants in the control arm were allocated to risk groups based on the RCE risks at baseline. GLMs were fitted with arm, level of risk and their interaction added as factors.

### Health economics

The costs of routine screening were measured using a mixed micro-costing and observational health economics analysis over a 2 year time horizon (see ESM Methods, Costs). Societal costs, including participant and companion costs, collected using a bespoke questionnaire, comprised time lost from work (productivity losses) and travel and parking costs. A detailed workplace analysis, measuring resources and staff time to deliver the screening programme, was conducted at each screening centre. This ingredient-based, bottom-up approach enabled a current resource-based cost to be attributed to the cost of screening each individual, taking into account both attendees and the related costs of non-attendance. We estimated the additional costs of running the RCE using a screen population size of 22,000 (Liverpool). Treatment costs were excluded as the 2 year time horizon was felt to limit any inference that could be attributed to lifetime cost (see ESM Methods, Costs).

A sample of the first participants enrolled into the RCT (*n* = 868) completed the EuroQol Five-Dimension Questionnaire (EQ-5D) five-level version (EQ-5D-5L) [21] and the Health Utilities Index Mark 3 (HUI3) [22] questionnaire at baseline and follow-up visits. Health state utilities were mapped [23] from the EQ-5D-5L to the EQ-5D three-level version and used a UK population tariff [24]. We applied a relevant Canadian tariff [25] to health state classifications of the HUI3 in the absence of an English or UK valuation set. Discounting was not applied, as both costs and QALYs were assumed to be assigned and incurred on an annual basis. Further detail is available in ESM Methods, Utilities and quality-adjusted life-years.

A detailed description of the cost-effectiveness analysis is available in ESM Methods, Cost-effectiveness methodology. A 90 day attendance window was utilised with a further 90 days added at 24 months to allow for the compounding lag in scheduling (see ESM Figs 1 and 2). We conducted multiple imputation of chained equations using available case data and followed guidance for best practice [26]. QALYs were derived using AUC, and incremental effects were estimated through ordinary least squares regression (for the univariate distributions of complete cases) and seemingly unrelated regressions (for the joint distributions of multiply imputed sets) on baseline utilities. We present unadjusted estimates as sensitivity analyses. We bootstrapped these regressions to characterise sampling distributions and derive 95% bias-corrected CIs around trial arm means and mean differences [27]. Intention-to-treat analyses were conducted in Stata/SE (Release 16; StataCorp, USA) from an NHS/societal perspective, and post-multiple imputation analyses followed Rubin’s combination rules for estimation within multiply imputed sets [28].

## Results

**Fig. 1:**
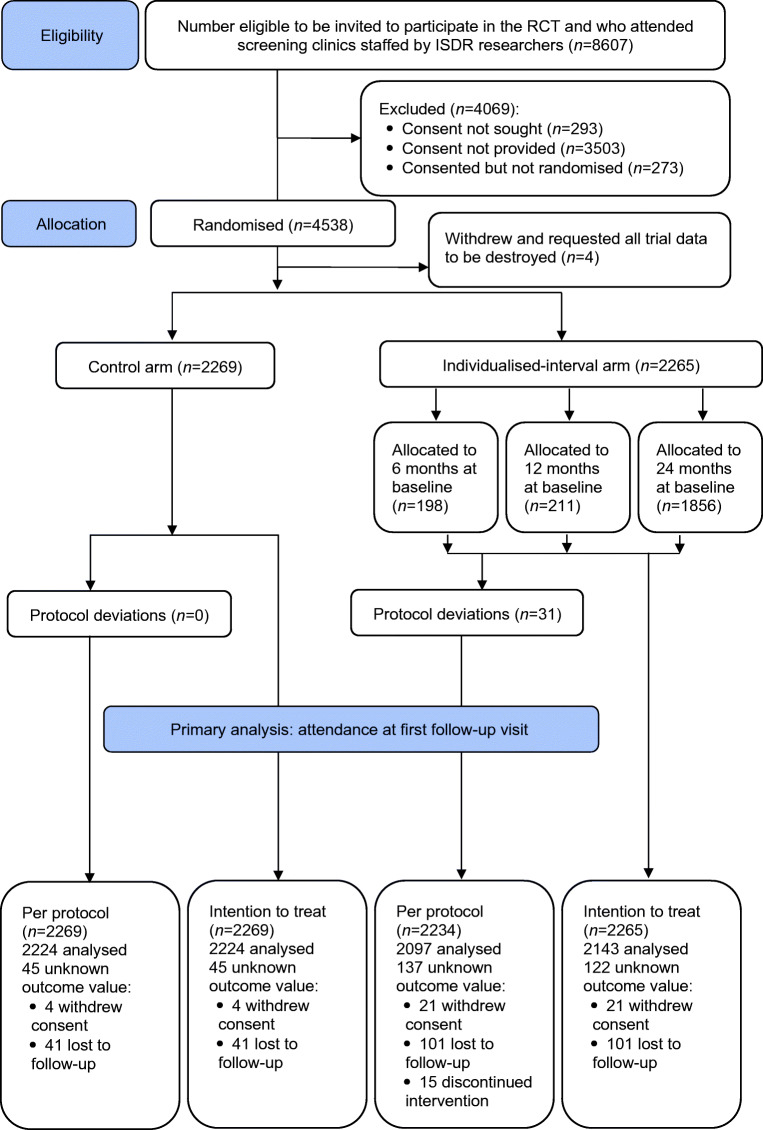
CONSORT 2010 flow diagram

Figure 1 summarises the trial profile showing the numbers for eligibility, allocation and withdrawals, and per-protocol and intention-to-treat datasets for the primary analysis. From 1 May 2014, 4538 participants were enrolled; four withdrew after randomisation, requesting removal of their trial data. Reasons for non-consent are shown in ESM Table 1. Allocations were 2269 to the control arm and 2265 to the individualised-interval arm (198, 211 and 1856 in the high-, medium- and low-risk groups, respectively). Last follow-up was on 5 September 2018.

The baseline characteristics of participants in the per-protocol dataset are shown in Table 1 (similar distributions for intention-to-treat are shown in ESM Table 2). Participants were aged 14–100 years (median 63 years), 60.4% were male, 94.6% were white and 88.5% had type 2 diabetes. Compared with the other two risk groups, those in the high-risk group were more likely to have type 1 diabetes, had a longer diabetes duration and higher HbA_1c_, and were less likely to have ever smoked. Proportions with any retinopathy by group within the individualised arm were 99.5%, 79.1% and 3.9% for those allocated to screening at 6, 12 and 24 months, respectively.

**Table 1:** Participant baseline characteristics by arm and screening interval allocation in 4503 participants in the per-protocol dataset

Baseline characteristic	Arm		Baseline risk group^a^			Overall total
	Fixed (12 months)	Individualised	High	Medium	Low	
*n*^b^	2269	2234	197	211	1826	4503
Sex, *n* (%)						
Male	1358 (59.9)	1360 (60.9)	124 (62.9)	135 (64.0)	1101 (60.3)	2718 (60.4)
Female	911 (40.1)	874 (39.1)	73 (37.1)	76 (36.0)	725 (39.7)	1785 (39.6)
Ethnicity, *n* (%)						
White	2140 (94.3)	2120 (94.9)	180 (91.4)	204 (96.7)	1736 (95.1)	4260 (94.6)
Asian^c^	48 (2.1)	30 (1.3)	2 (1.0)	3 (1.4)	25 (1.4)	78 (1.7)
Black	40 (1.8)	43 (1.9)	6 (3.0)	3 (1.4)	34 (1.9)	83 (1.8)
Chinese	7 (0.3)	6 (0.3)	1 (0.5)	1 (0.5)	4 (0.2)	13 (0.3)
Other	25 (1.1)	29 (1.3)	8 (4.1)	0 (0.0)	21 (1.2)	54 (1.2)
Unknown	9 (0.4)	6 (0.3)	0 (0.0)	0 (0.0)	6 (0.3)	15 (0.3)
Smoking status, *n* (%)						
Smoker	419 (18.5)	364 (16.3)	26 (13.2)	39 (18.5)	299 (16.4)	783 (17.4)
Ex-smoker	877 (38.7)	899 (40.2)	69 (35.0)	76 (36.0)	754 (41.3)	1776 (39.4)
Non-smoker	965 (42.5)	967 (43.3)	102 (51.8)	96 (45.5)	769 (42.1)	1932 (42.9)
Unknown	8 (0.4)	4 (0.2)	0 (0.0)	0 (0.0)	4 (0.2)	12 (0.3)
Diabetes type, *n* (%)						
Type 1	80 (3.5)	99 (4.4)	38 (19.3)	14 (6.6)	47 (2.6)	179 (4.0)
Type 2	2024 (89.2)	1962 (87.8)	140 (71.1)	180 (85.3)	1642 (89.9)	3986 (88.5)
Unknown	165 (7.3)	173 (7.7)	19 (9.6)	17 (8.1)	137 (7.5)	338 (7.5)
Age (years)						
Observed, *n*	2269	2234	197	211	1826	4503
Median (IQR)	63.3 (55.0–71.0)	62.8 (54.8–70.3)	58.3 (49.9–66.2)	60.9 (53.4–69.8)	63.7 (55.9–70.8)	63.1 (54.9–70.7)
Range	14.1–100.7	15.4–91.3	17.5–86.8	15.4–86.8	16.8–91.3	14.1–100.7
Disease duration (years)						
Observed, *n*	2267	2231	197	209	1825	4498
Unknown, *n*	2	3	0	2	1	5
Median (IQR)	6.9 (4.2–10.9)	7.0 (4.2–11.2)	11.1 (7.3–16.1)	9.8 (6.3–13.7)	6.4 (4.0–10.1)	7.0 (4.2–11.0)
Range	0.6–66.4	1.0–44.7	1.2–44.7	1.1–37.2	1.0–39.1	0.6–66.4
HbA_1c_						
Observed, *n*	2269	2232	197	211	1824	4501
Unknown, *n*	0	2	0	0	2	2
mmol/mol						
Median (IQR)	51 (44–61)	52 (44–63)	67 (53–84)	58 (51–67)	50 (44–60)	51 (44–62)
Range	26–146	28–155	33–134	34–155	28–104	26–155
%						
Median (IQR)	6.8 (6.2–7.7)	6.9 (6.2–7.9)	8.3 (7.0–9.8)	7.5 (6.8–8.3)	6.7 (6.2–7.6)	6.8 (6.2–8.8)
Range	4.5–15.5	4.7–16.3	5.2–14.4	5.3–16.3	4.7–11.7	4.5–16.3
Systolic BP (mmHg)						
Observed, *n*	2268	2234	197	211	1826	4502
Unknown, *n*	1	0	0	0	0	1
Median (IQR)	130.0 (121.0–138.0)	130.0 (122.0–138.0)	130.0 (124.0–138.0)	132.0 (124.0–140.0)	130.0 (122.0–138.0)	130.0 (122.0–138.0)
Range	84.0–213.0	90.0–204.0	93.0–175.0	95.0–204.0	90.0–200.0	84.0–213.0
Diastolic BP (mmHg)						
Observed, *n*	2208	2180	193	201	1786	4388
Unknown, *n*	61	54	4	10	40	115
Median (IQR)	76.0 (70.0–80.0)	76.0 (70.0–80.0)	77.0 (70.0–80.0)	77.0 (70.0–80.0)	76.0 (70.0–80.0)	76.0 (70.0–80.0)
Range	46.0–140.0	46.0–130.0	54.0–105.0	57.0–130.0	46.0–110.0	46.0–140.0
Total cholesterol (mmol/l)						
Observed, *n*	2258	2224	196	209	1819	4482
Unknown, *n*	11	10	1	2	7	21
Median (IQR)	4.0 (3.4–4.7)	4.0 (3.4–4.7)	4.0 (3.4–4.9)	4.0 (3.4–4.6)	4.0 (3.5–4.7)	4.0 (3.4–4.7)
Range	1.4–8.1	1.8–9.7	2.0–9.0	2.2–7.6	1.8–9.7	1.4–9.7
Retinopathy level, *n* (%)^d^						
R0 R0	1857 (81.8)	1800 (80.6)	1 (0.5)	44 (20.9)	1755 (96.1)	3657 (81.2)
R1 R0	262 (11.5)	296 (13.2)	58 (29.4)	167 (79.1)	71 (3.9)	558 (12.4)
R1 R1	146 (6.4)	137 (6.1)	137 (69.5)	0 (0.0)	0 (0.0)	283 (6.3)

^a^Differences across the three baseline risk groups (high, medium and low) were investigated, with statistically significant associations observed for diabetes type (*p* < 0.0001; Cochran–Armitage test), retinopathy level (*p* < 0.0001; Fisher’s exact test) and age (*p* < 0.0001), disease duration (*p* < 0.0001), HbA_1c_ (*p* < 0.0001) and systolic BP (*p* = 0.0101) (all Jonckheere–Terpstra test). No statistically significant associations across the three baseline groups were observed for sex (*p* = 0.30) or ethnicity (white vs non-white, *p* = 0.06) (Cochran–Armitage test), smoking status (*p* = 0.07; Fisher’s exact test), or diastolic BP (*p* = 0.06) or total cholesterol (*p* = 0.80) (Jonckheere–Terpstra test)

^b^Participants randomised who did not withdraw or request all data to be destroyed

^c^Asian ethnicity group excludes individuals with Chinese ethnicity

^d^An additional five individuals with one eye were randomised into the trial (0.1%). Four had R0 (no diabetic retinopathy) in one eye and were randomised into the fixed arm (0.2% of those in the fixed arm), while one had R1 (background retinopathy) in one eye and was randomised to the individualised arm (<0.1% of those in the individualised arm) and allocated to 6 month follow-up (0.5% of those in the 6 months allocation)

A total of 182 (4.0%) participants withdrew from the trial before the first follow-up: 25 (0.6%) withdrew consent, 15 (0.3%) discontinued the intervention and 142 (3.2%) were lost to follow-up (ESM Table 3). Withdrawals of consent were higher in the individualised arm (0.9% vs 0.2%). Loss to follow-up was higher in the individualised arm (101 [4.5%] vs 41 [1.8%]), probably exacerbated by the longer follow-up period of 24 months in the low-risk group (81.9% of the individualised arm). A total of 15 participants prematurely discontinued the intervention.

**Fig. 2:**
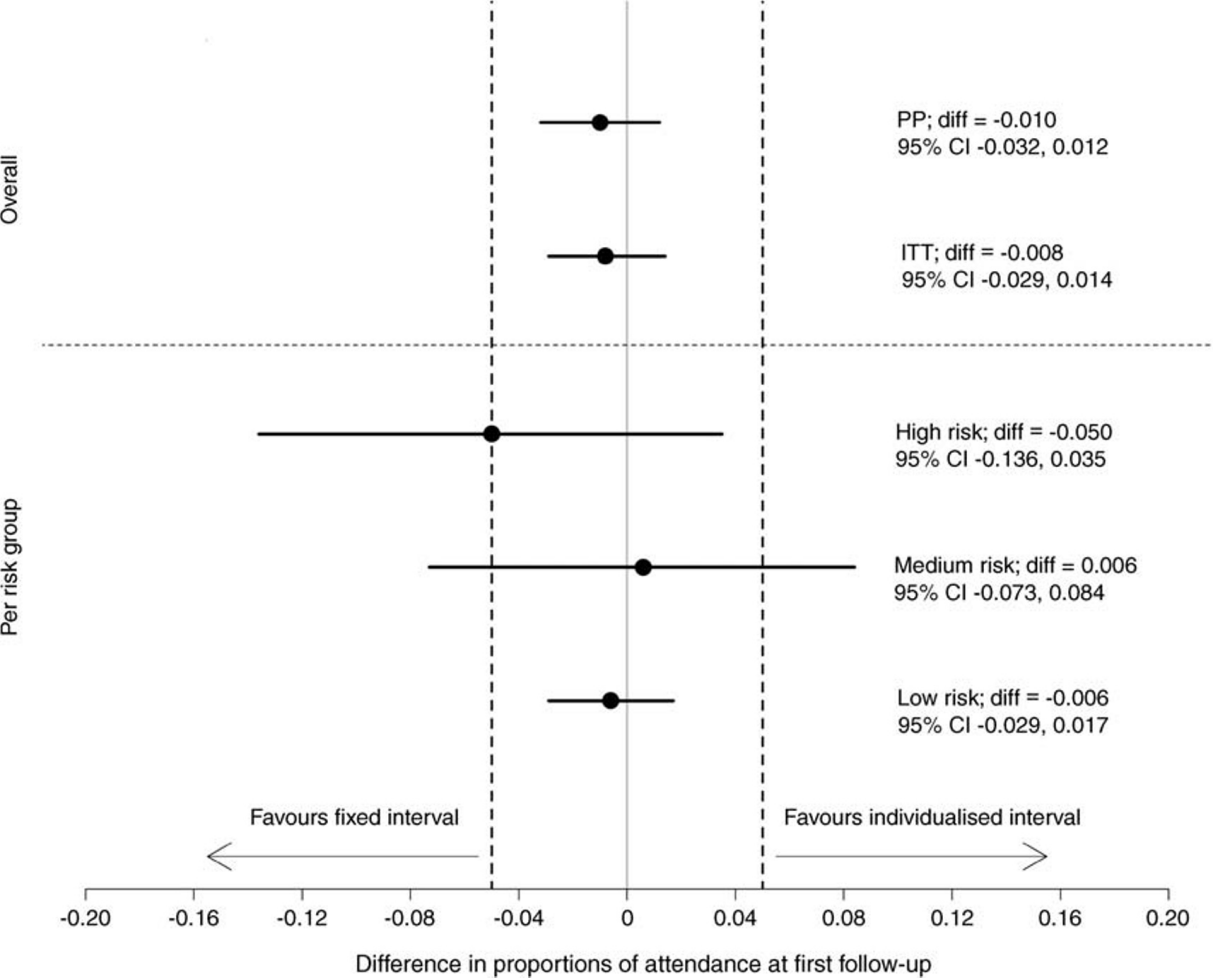
Difference in the proportion of participants attending the first follow-up visit between the two arms. ‘Overall’: primary analysis; ‘per risk group’: high, medium and low risk groups within the individualised arm. Point estimates and 95% confidence limits are provided. Vertical dashed lines indicate 5% predefined equivalence margin. Diff, difference; ITT, intention to treat; PP, per protocol

Attendance rates at first follow-up for the control and individualised arms were 84.7% (1883/2224) and 83.6% (1754/2097), respectively (difference in proportions −1.0 [95% CI −3.2, 1.2], per-protocol analysis). Against the predefined acceptability margin (5%), the two arms were regarded as equivalent (Fig. 2, Table 2). Protocol deviations resulting in exclusion from this analysis occurred in 31 participants in the individualised arm; no safety effect occurred (one participant was assigned to screening at 12 instead of 6 months and 30 were assigned to 6 or 12 months instead of 24 months). Similar results were obtained from the intention-to-treat analysis. Per-protocol and intention-to-treat analyses with multiple (Table 2) and simple (ESM Table 4) imputation confirmed equivalence in attendance rates between the two arms.

**Table 2:** Results of the test for equivalence in attendance rate at first follow-up visit and of non-inferiority in STDR detection within 24 months, based on the per-protocol, intention-to-treat and multiple imputation datasets

Outcome	Approach		Control arm			Individualised arm			Difference in proportions (*D* = *p*_*l*_ − *p*_*c*_)	95% CI^a^ of *D*	
			*n*	Attended/STDR detected	Proportion (*p*_*C*_)	*n*	Attended/STDR detected	Proportio*n* (*p*_*I*_)		Lower bound	Upper bound
Primary: attendance at first follow-up	Per protocol	Overall	2224	1883	0.847	2097	1754	0.836	−0.010	−0.032	0.012
		High risk	203	157	0.773	195	141	0.723	−0.050	−0.136	0.035
		Medium risk	169	138	0.817	208	171	0.822	0.006	−0.073	0.084
		Low risk	1852	1588	0.857	1694	1442	0.851	−0.006	−0.029	0.017
		Multiple imputation	2269	1910	0.842	2234	1870	0.837	−0.005	−0.026	0.017
	Intention to treat	Overall	2224	1883	0.847	2143	1798	0.839	−0.008	−0.029	0.014
		Multiple imputation	2269	1913	0.843	2265	1903	0.840	−0.004	−0.025	0.018
Secondary: STDR within 24 months	Per protocol	Overall	2042	35	0.017	1956	28	0.014	−0.003	−0.011	0.005
		High risk	176	20	0.114	127	17	0.134	0.020	−0.053	0.100
		Medium risk	157	5	0.032	179	7	0.039	0.007	−0.038	0.051
		Low risk	1709	10	0.006	1650	4	0.002	−0.003	−0.009	0.001
		Multiple imputation	2269	39	0.017	2234	36	0.016	−0.001	−0.009	0.007
	Intention to treat	Overall	2042	35	0.017	2056	32	0.016	−0.002	−0.010	0.006
		Multiple imputation	2269	39	0.017	2265	39	0.017	−0.001	−0.008	0.008

^a^Newcombe score (based on Wilson score) CIs

The equivalence margin was predefined as δ = 0.05 and the non-inferiority margin as δ = 0.015. Proportions in both analyses (i.e. of attendance and STDR detection) are denoted as *p*_*C*_ and *p*_*I*_ for the control and individualised arms, respectively. In both cases, results by risk group for the per-protocol approach are also included

Figure 2 and Table 2 show the equivalence analysis within the individualised arm. Equivalence in attendance rates at the first follow-up visit was found for the low-risk group (control 85.7%, individualised 85.1%, difference −0.6% [95% CI −2.9, 1.7]). For the medium-risk group, the difference in attendance rates was also very small (control 81.7%, individualised 82.2%, difference 0.6% [95% CI −7.3, 8.4]); however, equivalence was not confirmed due to the relatively wide CI. Attendance rates were lower in the high-risk group (control 77.3%, individualised 72.3%, difference −5.0% [95% CI −13.6, 3.5]) and equivalence was not observed. The attendance rates observed over 12 months (≥1 attended appointment), however, were higher in the individualised arm (89.1%) compared with the control arm (77.3%). A post hoc analysis of attendance over 24 months gave similar results (ESM Table 5).

The mean number of appointments per person by baseline risk allocation over 24 months was 1.83, 1.06 and 0.85 in the high-, medium- and low-risk groups, respectively. At least one change in allocation from baseline was recorded as follows: high-risk group, 48/160 (30.0%) participants were changed to a longer screening interval; medium-risk group, 34/200 (17.0%) participants were changed to a shorter screening interval and 84/200 (42.0%) were changed to a longer interval; low-risk group, 142/1694 (8.4%) were participants changed to a shorter interval (ESM Table 6). Overall, 132 participants were switched to a longer screening interval and 176 to a shorter interval.

There was no evidence of a loss of ability to detect STDR over 24 months from baseline in the individualised arm (28/1956; 1.4%) compared with the control arm (35/2042; 1.7%), with a difference of −0.3 (95% CI −1.1, 0.5) (Table 2). Non-inferiority was found within the low-risk group (control 0.6%, individualised 0.2%, difference −0.3 [95% CI −0.9, 0.1]). Non-inferiority was not confirmed for the high- and medium-risk groups, probably because of small participant numbers. Similar results were obtained with intention-to-treat and multiple and simple imputation analyses.

Four participants required treatment within 6 months of being screen-positive: two for STDR (one in the control arm and one in the high-risk group) and two for reasons other than diabetic retinopathy.

Withdrawals, premature discontinuations and loss to follow-up within 24 months showed a similar distribution across the arms (ESM Table 7).

Further safety data are presented in ESM Results, Secondary safety outcomes, and ESM Table 8. We did not detect a clinically significant worsening of diabetes control or an increase in visual impairment when comparing the groups.

As a secondary outcome, we investigated the efficacy of individualised screening using data on numbers of attended appointments and rates of screen-positive events across 24 months (ESM Table 9). Overall, 43.2% fewer screening attendances were required in the individualised arm vs the control arm (2008 vs 3536). Higher rates of screen-positive events by screening episode attended were seen in the individualised arm (individualised 5.1% [102/2008], control 4.5% [160/3536]). Within the individualised arm, the high-risk group had the highest screen-positive rate (high 10.7% [34/317], medium 6.0% [15/249], low 3.7% [53/1442]). In the high-risk group, most of the screen-positive results were because of eye disease other than diabetic retinopathy; the rate of participants who were screen-positive for diabetic retinopathy was low, at 0.5% (7/1442). Screening episodes that detected STDR were earlier in the individualised compared with the control arm: 6–12 months 17.9% (5/28) vs 2.9% (1/35); 12–18 months 32.1% (9/28) vs 60.0% (21/35).

**Fig. 3:**
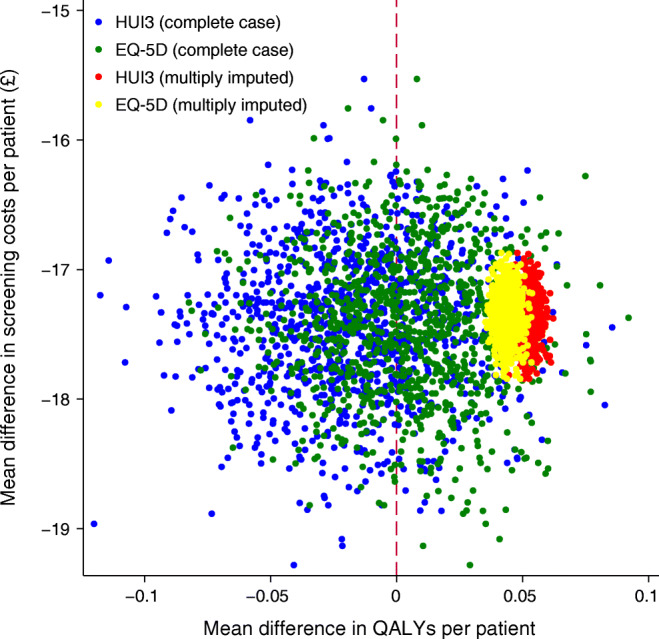
Baseline-adjusted EQ-5D, HUI3 and NHS perspective incremental cost-effectiveness of individualised vs annual screening from 1000 iteration bootstraps

A total of 868 participants completed the health economics questionnaires. ESM Table 10 presents the summary costs (2019/2020 values) associated with the screening programme. The cost to the NHS was £28.73 per attendance and £12.73 per non-attendance, while additional productivity losses and out-of-pocket payments by the patient accounted for £9.00. Within-trial summary health economic and cost-effectiveness data over the 2 year time horizon are reported in Table 3, and additional data for the two arms in ESM Tables 11 and 12. Multiple imputation supported the strict dominance of individualised screening in terms of QALYs gained and cost savings. Here, we briefly summarise the results reporting conservative data from an analysis of complete case QALYs and multiple imputation costs. Mean incremental QALY scores did not show a statistically significant difference between the trial arms (EQ-5D 0.006 [95% CI −0.039, 0.06], EuroQol Visual Analogue Score [EQ-VAS] 0.004 [95% CI −0.049, 0.052] and HUI3 −0.017 [95% CI −0.083, 0.04]; Table 3), with agreement between societal preferences (EQ-5D/HUI3) and individual preferences (EQ-VAS). Incremental cost savings per participant with individualised screening were: NHS perspective £17.34 (95% CI 17.02, 17.67); societal perspective £23.11 (95% CI 22.73, 23.53); corresponding to a reduction in total programme costs of 20% (from £193,983 to £154,386) and 21% (from £248,114 to £195,348), respectively. The individualised arm showed incremental savings across all domains. The NHS perspective cost-effectiveness plane for the EQ-5D and HUI3 shows the dominance of the intervention arm in cost savings and expected maintenance of quality of life (Fig. 3). While the intention had been to report cost-effectiveness acceptability curves, the dominance in cost reduction of risk-based screening and little fluctuation in QALYs across all instruments rendered this metric uninformative, as the proportion cost-effective was inelastic to varying thresholds. See ESM Results, Health economics for further details.

**Table 3:** Within-trial intention-to-treat QALYs and costs per participant: individualised vs annual screening

Variable (*n*/*N*)^a^	Mean difference (95% CI)^b^	
	Complete cases	Multiple imputed
EQ-5D (539/868)		
Unadjusted	0.012 (−0.097, 0.119)	0.043 (0.032, 0.055)
Baseline adjusted	0.006 (−0.039, 0.06)	0.044 (0.038, 0.05)
EQ-VAS (548/868)		
Unadjusted	−0.033 (−0.109, 0.044)	0.013 (0.005, 0.022)
Baseline adjusted	0.004 (−0.049, 0.052)	0.022 (0.017, 0.028)
HUI3 (408/868)		
Unadjusted	−0.016 (−0.135, 0.116)	0.068 (0.056, 0.081)
Baseline adjusted	−0.017 (−0.083, 0.04)	0.051 (0.045, 0.058)
Costs (4389/4534) (£)		
NHS perspective	−17.44 (−18.57, −16.31)	−17.34 (−17.67, −17.02)
Societal perspective^c^	−23.26 (−24.65, −21.92)	−23.11 (−23.53, −22.73)

^a^*n* corresponds to the number of univariate complete cases out of the sampled set size of *N*

^b^We estimated 95% CIs through 1000 iteration bootstrap regressions for univariate distributions of complete cases, and seemingly unrelated regressions for multivariate distributions of multiple imputed sets

^c^Societal costs report the combination of NHS costs, participant or carer productivity losses, and out-of-pocket expenses

## Discussion

Our study shows that individualised, risk-based, variable-interval screening appears to be safe. Attendance at the first follow-up visit was equivalent to that for annual screening and secondary safety findings on the detection of STDR, visual function and glycaemic control are supportive. Our approach reduced the number of appointments by more than 40%. There were no detectable effects on quality-of-life measures and convincing cost savings.

Important strengths of our study are its RCT design, size, independent oversight and direction from an expert PPI group. We used the emerging technology of risk calculation, based on integrating local clinical data in estimating risk, to introduce a personalised approach. We implemented a mixed Markov RCE into a clinical trial setting. Protocol deviations were few (1.4% for primary outcome) and there were only moderate numbers of withdrawals and loss to follow-up, which are inevitable in a large RCT such as this. Findings were similar in our per-protocol, intention-to-treat and multiple imputation analyses, which followed current guidance on equivalence studies [20].

Generalisation of our findings has some limitations. Participants were enrolled from a single programme that has been running for more than 30 years, and consequently had relatively low rates of baseline diabetic retinopathy and progression to STDR. Good glycaemic and BP control and a relatively low proportion of participants with type 1 diabetes (4.0%) might have biased the sample. Low rates of diabetic retinopathy have been reported in other similar settings [29, 30], but our results should be treated with caution in areas with a higher prevalence, poorer control of diabetes or wider ethnic group representation, or in programmes during the set-up process.

Our trial had only a 2 year time horizon, which is short in the context of a life-long condition. With a move to extended-interval screening in several countries, we wanted to provide high-quality RCT evidence on how people act when given risk-based, variable-interval screening. Previous studies have derived risk models and used them in validation studies but have not answered this question [31, 32]. To allow for two cycles for the low-risk group would have extended the study duration from 4 to 6 years. The low rates of retinopathy and STDR in the low-risk group suggest that our findings on effectiveness would be unlikely to change; most disease was detected in the high- and medium-risk groups (STDR by 24 months: high-risk group 37 [12.2%], medium-risk group 12 [3.6%], low-risk group 14 [0.4%]). The cost savings with variable-interval screening were substantial and are unlikely to be lost over a longer time horizon, but to continue and accrue year on year. Robust monitoring including fail-safe mechanisms, such as in our design, should be included in any future implementation of risk-based, variable-interval screening.

A move to longer screening intervals for people at low risk of diabetic retinopathy has previously been suggested [33–35], but without convincing evidence on safety [10]. Overall, 19.0% of people invited to take part in our study explicitly stated that they wished to remain on annual screening or did not want a change of interval (ESM Table 1). Health professionals fear that extending screening intervals may reduce perceptions of the importance of screening, leading to loss of engagement and worse diabetes care. We did not detect a worsening in glycaemic control. Our findings give substantial reassurance that a 24 month interval for a low-risk individuals with diabetes in a setting such as ours is safe. However, for resource-poor or rural settings in low- and middle-income countries, further research is required before longer intervals can be contemplated.

Using a risk-based approach allows personalisation, offering better targeting of high-risk groups and improved patient engagement. Around 15% of participants in this study had at least one change in their risk-based interval; 59% (118/200) of those allocated to attend annual screening (the current standard) experienced at least one change of interval (ESM Table 6). Aspelund and colleagues have developed a similar risk engine for diabetic retinopathy screening using modelling coefficients from the 1990s [36] and conducted external validation in a Dutch cohort [31]. The strength of our RCE is that it was populated with local data and can be regularly updated with current data to reflect changing local progression rates.

For our approach to be more widely adopted, assessment in other local and national screening programmes will be required. Some systems development from our research setting to an implementation environment will be required. Our parallel social science study demonstrated the acceptability of variable-interval, risk-based screening to individuals with diabetes and health professionals, provided that additional monitoring and fail-safe mechanisms are included [37]. Further evaluation should include the effect of factors such as the unexplained heterogeneity among screening programmes in England in terms of grading outcomes and screening uptake.

We targeted high-risk people using a 6 month screening interval. Attendance rates in this group were lower in the individualised arm (72.3%) compared with the control arm (77.3%), but the shorter interval allowed more frequent screening and earlier detection of disease. There were higher relative rates of STDR detection in the high- and medium-risk individualised groups (13.4% and 3.9%) compared with 1.7% in the control arm, with very low rates in the low-risk group (0.2%). Our study was powered for equivalence with all risk groups combined and not for the risk group comparisons. Despite the hypothesis of equivalence not being supported in the high-risk group, the attendance rates observed over a period of 12 months were considerably higher in the individualised arm compared with the control arm (89.1% vs 77.3%).

Including systemic clinical risk factors (HbA_1c_, systolic BP, lipids) adds value in several ways. It allows the introduction of a high-risk group with earlier detection of STDR. It also improves patient engagement by linking retinopathy to systemic control; our PPI group strongly advised that including clinical data reinforces the message that control is crucial in managing complications. Including systemic risk factors also improves the accuracy of identifying low-risk patients when compared with simpler stratification strategies as suggested for the UK [9], while maintaining a desirable level of sensitivity. A post hoc analysis of our RCT dataset estimated that a simpler stratification approach [9] would allocate 66.9% of participants at baseline to a 24 month screening interval (compared with 81.9% in ISDR) and 33.1% to 12 months (data not shown). We have observed that multivariate risk models tend to require a lower frequency of eye examinations, and consequently are likely to be more cost-effective than current care [38].

Adding systemic risk factors may not be feasible in many settings where it has proved difficult to reliably link primary and secondary care data because of issues with data ownership and IT system management. We overcame this through strong support from local health commissioners and primary-care research groups. We needed to develop bespoke data processing, imputation and data validation processes. We included this in our cost-effectiveness analysis.

Our data show convincing evidence that an individualised approach provides considerable cost savings compared with annual screening. In addition, moving to variable-interval, risk-based screening did not compromise participants’ quality of life. Incremental screening cost savings of £17.34 were achieved (NHS perspective), rising to £23.11 (societal perspective) per participant over the 2 years, a reduction in total programme costs of 20% and 21%, respectively. A key driver in achieving cost-effectiveness was the reduction in unnecessary appointments and efficient use of administrative time. In a screening population such as in Liverpool (22,909 invitations in 2018–2019), this may amount to annual savings in the region of £199,000. In England (screening population 2.76 million [2018–2019] [39]), this could amount to around £23.9 million in annual savings for the NHS, rising to £31.9 million from a societal perspective. Such resources could be used to target groups that are hard to reach and those at high risk of visual impairment, and to more cost-efficiently screen the expanding population of individuals with diabetes. Furthermore, those in low-risk groups would be spared the inconvenience and additional personal cost of attending superfluous appointments.

The large number of observations and the accuracy of the true resource cost of screening are strengths of our cost-effectiveness analysis. The work could have been further strengthened by taking a long-term time horizon as discussed above and including the costs of treatment and blindness averted. Collecting quality-of-life data from every participant in the study would also have strengthened the analysis; our sample size was chosen to minimise disruption in the screening clinic. While methods of multiple imputation involve varied assumptions, the agreement between our complete case and multiply imputed quality-of-life data, across instruments and adjustments, is encouraging in viewing individualised screening as a cost minimiser (see supporting discussion in ESM Discussion).

Our data on efficacy show a higher efficiency of the individualised approach, with a greater proportion of screening episodes being positive (5.1% vs 4.5% in the control arm). A number of benefits include a lower burden of appointments, earlier detection of STDR for people at high-risk and an increased capacity to see individuals who have been newly diagnosed with diabetes. Furthermore, in the era of personalised care, a shortened screening interval in people at high risk might increase the focus on risk factor control and engagement with screening.

For people identified by our RCE as being at low risk, the rates of screen-positive for diabetic retinopathy were very low at 0.5% and even lower for STDR at under 0.2%. This is likely to apply elsewhere, but should not be applied in territories without an established systematic screening programme, where the first-pass prevalence will be high. The design of our study and the concerns around safety restricted us to a maximum screening interval of 24 months. However, our data suggest that extending intervals beyond 2 years would be reasonable.

In conclusion, our study, the largest RCT performed to date in ophthalmology or screening, should reassure all stakeholders in diabetes care that extended and personalised interval screening can be safely and effectively introduced in established systematic screening programmes. Our evidence is over a 2 year time horizon, so for implementation the long time frame of the disease should be addressed by continuous monitoring of attendance, retinopathy rates and grading quality. It is also applicable to other settings where clinical data are available, such as in healthcare-delivery organisations and polyclinics. Where current recommendations are for annual screening, we provide evidence to support a move to variable intervals with substantial reductions in cost.

## Supplementary Information

ESM (PDF 877 kb)

## Data Availability

A fully anonymised dataset with supporting data dictionary will be available from the corresponding author 3 months after the publication date for 3 years to recognised research institutions subject to approval by the ISDR Data Governance Committee of an analysis plan, a data access agreement, appropriate acknowledgement and funding for additional costs. Results will be disseminated to patient organisations.

## Abbreviations

EQ-5D: EuroQol Five-Dimension Questionnaire
EQ-5D-5L: EuroQol Five-Dimension Questionnaire five-level version
EQ-VAS: EuroQol Visual Analogue Score
GLM: Generalised linear model
HUI3: Health Utilities Index Mark 3
ISDR: Individualised Screening for Diabetic Retinopathy
NHS: National Health Service
PPI: Patient and public involvement
QALY: Quality-adjusted life-year
RCE: Risk-calculation engine
STDR: Sight-threatening diabetic retinopathy
