# Supplementary material for: Safety and cost-effectiveness of individualised screening for diabetic retinopathy: the ISDR open-label, equivalence RCT
Source: Diabetologia. 2020 Nov 4;64(1):56–69. doi: 10.1007/s00125-020-05313-2 (PMC7716929; doi:10.1007/s00125-020-05313-2)
Supplement: Supplementary file 1 — (PDF 877 kb) [file 125_2020_5313_MOESM1_ESM.pdf]

Electronic Supplementary Material

Broadbent et al, Safety and cost effectiveness of individualised screening for diabetic retinopathy: the Individualised Screening for Diabetic Retinopathy (ISDR) single centre, open label, equivalence RCT

TABLE OF CONTENTS

ESM METHODS 2

Clinical Trials Research Centre (CTRC) procedures 2

Trial management 3

Secondary outcomes 3

Sample size 4

Costs 4

Utilities and quality-adjusted life-years (QALY) 4

Cost effectiveness methodology 5

ESM RESULTS 6

Secondary safety outcomes 6

Health economics 7

ESM DISCUSSION – HEALTH ECONOMICS 7

Supplementary references 7

ESM Table 1. Reasons for consent not being provided 8

ESM Table 2. Participant baseline characteristics by arm and screening interval allocation in 4534 participants in the ITT dataset 10

ESM Table 3. Withdrawals of consent, premature discontinuation of intervention and lost-to-follow-up by arm prior to first follow-up visit (primary analysis; attendance) 12

ESM Table 4. Tests for equivalence in attendance rate at 1st follow-up visit and for non-inferiority in STDR detection within 24 months, based on simple imputation of the per-protocol and intention-to-treat datasets. 13

ESM Table 5. Test for equivalence in attendance proportion over 24 months based on the per-protocol dataset (post hoc analysis) 13

ESM Table 6. Breakdown of the direction (increase/decrease) of intervals at first switch from baseline allocation over 24m by risk group in the individualised arm 14

ESM Table 7. Withdrawals of consent, premature discontinuation of intervention and lost-to-follow-up by arm within 24 months 14

ESM Table 8. Descriptive table summarising secondary outcomes by arm 15

ESM Table 9. Numbers of attended follow-up appointments and screen positive events within 24 months (+90 day window) by arm using the per-protocol dataset at 24 months 17

ESM Table 10. Micro-costing of the Liverpool Diabetes Eye Screening Programme and estimated costs of the Risk Calculation Engine 18

ESM Table 11. Control arm summary statistics of intention-to-treat health economic data at 24 months . 19

ESM Table 12: Individualised arm summary statistics of intention-to-treat health economic data at 24 months 20

ESM Fig 1. Control arm distributions (frequency) of scheduled screening appointments 21

ESM Fig 2. Individualised arm distributions (frequency) of scheduled screening appointments 21

MEMBERS OF THE ISDR STUDY GROUP NOT QUALIFYING AS AUTHORS 22

ESM METHODS

Clinical Trials Research Centre (CTRC) procedures

i) Data collection and handling

The Individualised Screening for Diabetic Retinopathy (ISDR) randomised controlled trial (RCT) used a combination of data collection methods. Source data collected on paper clinical report forms (CRFs) were received centrally from the screening centres or the hospital eye service clinics by the ISDR data managers. A freeware software package designed for clinical trial data entry, Open Clinica (OC), received CRF data and systemic clinical data from the purpose built ISDR data warehouse (DW).

The ISDR trial data flowed electronically between the multiple data sources including the DW, OC, randomisation system and the risk calculation engine (RCE). Transfer of electronic data occurred within 24 hours of the scheduled visit and was considered source data for the trial unless specified differently. Allocations from the RCE were received electronically into the Liverpool Diabetes Eye Screening Programme (LDESP) management system (OptoMize, EMIS Health, UK) for generation of participant letters.

Validations were programmed into the ISDR OC system. The Statistical Team Leader, Database Designer and Data Manager or Senior Data Manager were the primary personnel involved in proposing, reviewing and amending these automated checks. These automated validations were designed to raise warnings at the point of data entry should data appear to be incorrect, out of range or invalid. Data queries were either manually raised by the OC user or automatically where data were missing or where warnings were flagged by the system.

SDV refers to source data (document) verification and normally involves checking source data against data recorded on CRFs in order to ensure quality of the trial data. For ISDR the SDV referred to comparison of data on CRFs with that entered on the database.

All (100%) of primary outcome data for randomised patients were checked against CRFs to ensure accuracy of entry.

Unknown attendance comprised withdrawal of consent, premature discontinuation of the intervention or loss to follow-up. Sight-thtreatening diabetic retinopathy (STDR, DR) status was recorded as unknown if withdrawal occurred prior to 24 months. Intention-to-treat (ITT) analyses including every participant according to assigned randomisation were also conducted for sensitivity analyses.

Numbers and % described categorical variables, and mean (SD), or median (interquartile range (IQR)) (if deviations from normality were detected) described continuous variables. Comparisons between arms for VA were conducted using the Mann Whitney U test. There was no allowance for multiplicity. All confidence intervals (CIs) were two-sided. SAS (v9.3) was used for the statistical analyses.

ii) Operational bias

Operational bias was minimised using the following processes:

- Automatic validations (as described above) to fire warnings at the point of data entry should data appear to be incorrect, out of range or invalid.
- Source data verification by checking the data on CRFs against that entered on the database to assure accuracy of data entry.
- A risk assessment was followed to look at safety risks from clinical procedures specified by the protocol, risks related to participant rights, risks to the reliability of trial results and organisational risks. This process was used to identify potential vulnerabilities in trial design and methodology, form a common understanding by all stakeholders on the risks for the trial, facilitate a risk proportionate approach to the trial activities, including the regulatory requirements and developing the trial management and monitoring strategies.
- The allocation sequence was generated by an independent CTRC statistician (someone different to the trial statistician and quality control (QC) statistician)

- A statistical analysis plan (SAP) was developed to describe the pre-planned analyses and reporting. SAPs were a requirement for Independent Data and Safety Monitoring Committee (IDSMC) reports, including a closed section where allocation groups were used, and for final analyses, included reports restricted to descriptive analyses.
- A “blind review”, just before data lock, by the trial statistician where the overall (not by arm) summary statistics for the primary and secondary outcomes were assessed to detect any potential data errors
- A QC statistician (independent to the trial statistician) performed independent programming for the primary outcome and safety outcomes

iii) Withdrawals and missed appointments
Three levels of withdrawal were defined: withdrawal of consent (option to have all data previously recorded in the trial retained or removed), premature discontinuation of intervention (reverted to annual screening), lost to follow-up (true screen positive (STDR) and consequently transferred to the hospital eye service; moved to a different area; died; pregnancy; other). Participants who did not attend their first appointment were sent a second. If they failed to keep this they were recalled in the next round of screening (6 months for the high-risk group, 12 months for medium, low-risk and control).

Trial management
A Trial Management Group met bi-monthly reporting to the IDSMC which met annually and gave safety recommendations to the Trial Steering Committee. All committees had patent and public involvement (PPI) representation. The IDSMC undertook annual review of the trial progress by:
- assessing data quality, including completeness
- monitoring recruitment figures and losses to follow-up
- monitoring compliance with the protocol by participants and investigators
- monitoring evidence for treatment differences in the efficacy and safety outcome measures
- recommending or advising on any major changes to the protocol, where necessary (e.g. changes to the recruitment procedures, inclusion criteria, endpoints, data collection, etc.)
- requesting additional data analyses for monitoring purposes of the IDSMC
- assessing the impact and relevance of any external evidence provided

There were no stopping rules in the study, however if the IDSMC or Trial Steering Committee had any concerns with data or safety they could recommend that the study be stopped. CTRC statisticians undertook the analysis for each IDSMC report. There were no amendments other than further clarification on information and additional details in the report made by the data management committee. The Committee met on an annual basis as agreed at the first meeting and in line with recruitment and follow-up data.

Secondary outcomes
Due to the nature of ISDR, some outcomes refer to both safety and efficacy, and the line between the two is not easy to draw. For example detection of STDR provides evidence of safety through disease missed and efficacy through numbers of screen episodes required to detect STDR. STDR was a key patient related outcome identified by the patient expert group. We report secondary outcomes (listed on page 20 of the protocol) in Supplementary Table 9.

Three secondary endpoints: new visual impairment due to DR, number of dedicated diabetes assessment clinic appointments and the number of other eye appointments for DR, were not assessed in the final analysis as described in the SAP. These three variables refer to true screen positive patients. True screen positive patients are "withdrawn" from the trial and consequently these variables were not available to be analysed.

In addition, the following data (listed on page 31 of the protocol) proved very difficult to collect:
- number of dedicated diabetes assessment clinic appointments (biomicroscopy)
- number of other eye appointments for DR
- hospital attendance for diabetic life-threatening events (heart attack and stroke) in both arms of the trial.
- attendance at GP annual review rates in both arms of the trial.

For the outcome “STDR within 24 months” reported in ESM Table 9, any individual who withdrew, prematurely discontinued the intervention or were lost-to-follow-up (except STDR cases) prior to 24 months post-baseline were considered to have an unknown STDR outcome. This is because they were not in the trial at 24 months and could have developed STDR by that time.

For the outcomes retinopathy level, maculopathy level, visual acuity and visual impairment values were taken from the last attended follow-up within 24 months (allowing for a 90-day window for attendance). In these cases, any individuals who withdrew, prematurely discontinued the intervention or were lost-to-follow-up (except those who were screen positive at the last follow-up appointment) prior to the end of the 24-month follow-up period were considered to have an unknown outcome value.

For the outcome screen positive within 24 months, any individual who withdrew, prematurely discontinued the intervention or were lost-to-follow-up (except screen positive cases) prior to 24 months post-baseline were considered to have an unknown screen positive outcome. This is because they were not in the trial at 24 months and could have been screen positive by that time.

For the outcome "false positive within 24 months", out of those defined as being screen positive within 24 months, any individual who did not attend a biomicroscopy for any reason were considered to have an unknown false positive outcome.

Sample size

Due to a slower recruitment rate than expected, the sample size required was revisited in November 2015. We considered a range of possible values for the recruitment rates based on national estimates of attendance rates, which were close to 75% and on recent figures of attendance observed at the time in the Liverpool population (minimum value just below 75%). We were aware that very small deviations from the initial 75% expected recruitment rate, for example $\pm$0.4%, would result in differences in sample size of about $\pm$90 patients, and consequently decided to adopt a conservative value for the expected rate to cover a range of possible scenarios. We used Equation (9.12) [1] with allocation ratio φ=1, 10% two-sided type II error ($z_{0.95}$, power=90%), 2.5% one-sided type I error ($z_{0.975}$), equivalence margin=0.05 and equal attendance rates $\pi_1=\pi_1=\pi=0.746$. The sample size obtained, after dividing by (0.94)^2 to allow for 6% annual loss over 24 months, was n= 4460. The equivalence margin 5% was regarded by the clinical investigators as a minimum difference in attendance rates acceptable for implementation of the risk-based variable-interval approach into clinical practice and based on experience in other equivalence and inferiority trials that have resulted in changes in clinical practice.

Costs

Across multiple screening sites, a mixed methods study examined the costs of screening for sight-threatening diabetic retinopathy within the Liverpool Diabetic Eye Screening Programme (LDESP). This involved identifying the processes and patient pathways associated with photographic screening, measuring resource use, estimating the average cost of resource use, and the average cost of each patient pathway. Data were retrieved from the ISDR clinical data warehouse of screening appointments attended by participants during the 2016 calendar year. Local resource use data were obtained from staff at the screening sites across the LDESP programme. The costing of resource use, such as the banding of NHS staff salaries, used unit costs derived from the Personal Social Services Research Unit unit costs of health and social care 2017.[2] The ISDR visit questionnaire, administered to the aforementioned 868 participants for whom we observed baseline health economic data, informed the cross-sectional study of productivity losses and out-of-pocket costs to participants and carers associated with the attendance at screening.

These costs were inflated at the midpoint of the NHS financial year; an increase of 4.373% from October 2017 to October 2019 specified by the inflationary rates of the Office for National Statistics[3] for Medical Services and Paramedical Services (CDID: D7FA). Following input from the trial management group and stakeholders, we estimated the additional costs of running the RCE using a screening population of size 22,000 (Liverpool).

Attendance records at screening appointments were near complete, from which we assigned the results of the costing study as unit costs. Unlike the estimates of NHS reference costs, which last reported a mean unit cost of £31 in 2010/11,[4] our method allowed a more granular characterisation of costing distributions whether patients attended, did not attend, or withdrew from screening, and without any additional patient burden that would normally be associated with this kind of data collection. For withdrawals, we assigned screening costs of £0 for those 110/4536 participants who died, were pregnant, or who were no longer diabetic and as such were no longer eligible for screening. If safety standards of screening are maintained, then the costs of NHS screening represent the differential cost driver of individualised versus annual screening intervals, assuming cost effective treatment further down patient pathways. External healthcare records were additionally considered to isolate treatments attributable to DR, however only a handful of these were observed during the within trial time horizon of two years. These treatments would have introduced bias and noise into our estimates and not represent the true differences in trial arm costs. Furthermore we do not capture the costs of confirmatory slit lamp biomicroscopy following a referable screen positive event due to the procedure taking place outside of the screening pathway. As a result our analysis slightly *underestimates* the propective cost savings of individualised screening versus usual care through a reduction in screening false positives. Further work which extrapolates towards a lifetime horizon should experience no such difficulty in including these treatments.

Utilities and quality-adjusted life-years (QALY)

Baseline health economic data were observed for the first 868 eligible individuals, out of a total intention-to-treat study size of 4534, yet it should be emphasised this was limited data collection by design. As such these data fulfil the criteria of missing completely at random (MCAR), where the likelihood of missingness is independent of unobserved data, and will not bias complete case analysis since the reason for the missing data is also unrelated to the outcome.[5,]

[6] Additionally, an unforeseen software error led a number of Health Utilities Index (HUI) classifications to be irretrievable – the affected observations were evenly split between trial arms and can similarly be considered MCAR. Health state utilities were derived from the EQ-5D, being the measure of choice of the National Institute for Health and Care Excellence (NICE), and the HUI3 for it's inclusion of a sight specific component, as such exhibiting greater sensitivity to diabetic retinopathy attributable changes in health related QoL. Following NICE's October 2019 position statement;[7] we mapped patients' item-level health state classifications, from the EQ-5D-5L instrument, to the 3L values using an appropriate mapping tool, the EuroQoL dataset, a Copula cumulative distribution model, and UK valuation set. Our estimations for the HUI3 applied a relevant Canadian tariff in absence of an English or UK valuation set. EQ-VAS estimates were divided by one hundred to aid comparison with the other instruments. Elicited utility scores of zero were input for 39 total participants when censored due to death. These solely represent individuals who supplied baseline health economic data, and not for those unobserved data which would have introduced an association between death and the observation of health state outcomes. Of note: these patients represent the number of zero value health state utilities reported at 6 months within our summary statistics. While not all patients were normally observed at this timepoint, we chose this method to best inform QALY construction and similarly this number, and those who later died, were amended at the 12, 18, and 24 month timepoints to ensure correct granularity. Similarly, observed utilities at each appointment were assigned to their nearest 6-month interval (allowing for 90 days on either side).

Area under the curve (AUC)[8] was used to adjust participant quality of life for the time spent in their respective health states and for constructing quality adjusted life-years (QALYs). It should be noted that patients completed health state utility instruments at baseline and at each follow-up visit, so there existed a differential in the frequency of observed responses between arm allocations at each appointment i.e. patients allocated to the high risk group at baseline completed the EQ-5D-5L and HUI3 at six months, the medium risk group at 12 months, and the low risk group at 24 months. This meant we observed greater intra-patient utility variation for higher risk participants since AUC assumes a linear relationship between health state utilities at each discrete time point. For robustness we conducted parallel analysis which constructed QALYs through change from baseline; however it had little effect on our estimates as the intra-variation in the utility of high risk patients was significantly outweighed by inter-patient variation. We utilised AUC in order to include all available data points in the generation of complete case QALYs, and because it allowed the use of far greater available case data within models of multiple imputation. Discounting was not applied as both costs and outcomes (QALYs) were assumed to be assigned and incurred on an annual basis.

Patient-level baseline utility imbalances between the control and individualised arms, regardless of statistical significance or missingness, would have led to inaccurate QALY estimates.[9] To control for this confounding, a binary variable representing the personalised arm, alongside a continuous variable for baseline utility, was regressed on total QALYs to estimate mean differences, and adjusted at means in our presented summary statistics. Means, standard deviations, and visual histogram plotting were used to compare complete versus available case utility data, exhibiting no systematic differences in values or distributions and so supporting the suitability of this method.[10] We present unadjusted estimates alongside as sensitivity analysis (covariate adjustment in addition to baseline adjustment had little effect on mean differences). The summary statistics we report are univariate distributions since deriving the multivariate distributions of complete case data of differential observational set sizes would necessarily exclude a significant number of observations from our larger QALY and costing sets.

Cost effectiveness methodology

Patient health state utilities were assigned to timepoints allowing for a 90 day attendance window (patients were allowed up to two additional appointment invitations if they did not attend their original scheduled appointment before reverting to usual care). However this presented difficulties at the 24 month timepoint as this window assumes that patients attended their scheduled appointments on the first day of the grace period of previous appointments. ESM Figs. 1 and 2 show the distribution of the first four scheduled screening appointments for patients in the control arm and individualised arm respectively, where we observe this compounding and significant lag in scheduling, frequently for those who missed earlier appointments. The scheduling of screening appointments incurs an irreversible cost, which will be accrued whether the patient attends or not. Consequently we allowed a maximum further 90 day attendance window for patients to capture these 24 month treatment-attributable costing distributions.

To control for unplanned missingness, assumed to be missing at random (MAR), methods of multiple imputation were applied as they build into their model the inherent uncertainty associated with the missing data: Multiple Imputation of Chained Equations (MICE) were performed using available case data, where a separate conditional distribution was specified for each imputed variable.[11, 12] Variable selection, excluding the EQ-5D, EQ-VAS, and HUI3 which must always be included as our outcomes of interest, was resolved in part through stepwise regression on utilities (forward selection due to the number of candidate variables excluding the possibility of backward elimination), manual testing of fit judged by Akaike Information Criterion (AIC), and logit modelling of missingness

mechanisms. We then examined variable covariance, in combination with the heterogeneity of variables across the total sample, to reduce overfitting and ensure model convergence.

We observed significant collinearity of health state utilities at six and eighteen months due to smaller sample sizes within the intervention arm, and as such excluded these timepoints from our model. However, we consider it unlikely to impact our estimates of mean differences: 1. This did not affect complete case observations. 2. affected patients, at worst, matched the granularity of those observed in the control arm for the purposes of AUC QALY construction. 3. Imputing at the index rather than QALY level avoided the greater bias of excluding a larger number of available case observations.

Final model specification ran fifty imputation sets (m50),[13] and included conditional functions of gender, smoking status, age, self-completion, presence of retinopathy in either one or both eyes at baseline or first followup,[14] and the EQ-5D, EQ-VAS, and HUI3 utilities across all appropriate time points. Following methods of best practice, multiple imputation was conducted individually for control and individualised arms respectively. Predictive mean matching (k-nearest neighbours (knn) 5) was utilised for utilities due to their non-normal distribution (heavy left tails) and bounded values. As costs presented minimal missingness and converted from participant attendance history, observations for the 145 participants who moved to a non-participant GP were imputed using predictive mass matching of total screening and societal costs.[15] We observed a slight difference in incremental cost between those who informed our QoL data, and those patients across the larger distribution set of screening costs, leading us to question the validity of our MAR assumption. After examination, these differences appear to be a relic of sample size – increased noise of screening behaviours in both arms - perhaps provoked by observation at the screening clinic. We considered it prudent to impute costs across the larger set excluding QoL data (imputation of utilities did include costs as a passive predictor). Logit functions of categorical variables were augmented to avoid the bias of perfect prediction.[16] The models were run multiple times, and QALY distributions (derived from multiply imputed utilities) were visually inspected to confirm the robustness of parameter estimates.

Between arm differences in arithmetic means are the parameters of interest within economic evaluations of randomised control trials.[17] Following multiple imputation we ran seemingly unrelated regressions (SUR)[18] to estimate incremental QALYs and costs; a simultaneous method which permits baseline and covariate adjustment, while capturing the multivariate distributions of our outcomes through allowing correlation between the error terms of our regressions. To reiterate our earlier disucssion – due to possible violation of MAR we present point estimates (and CIs) of multivariate distributions of utilities, and univariate distributions of costs. All analysis followed Rubins combination rules for estimation within multiply imputed sets.[19]

These SURs were bootstrapped, a nonparametric method to characterise sampling distributions (practical when these distributions do not hold a Gaussian form), to derive bias-corrected confidence intervals around the between-arm mean differences in QALYs and costs.[17, 20] We would like to particularly recommend the paper of Schomaker and Heumann for their work on "Bootstrap inference when using multiple imputation".[21] We opted for MI BOOT (pooled sample), at the cost of greater symmetry in our confidence intervals, due to the infeasibility of BOOT MI procedures regarding the large number of imputed sets and large sample size (months rather than days of estimation time).

Mean differences in QALYs and costs were used to construct incremental cost effectiveness ratios (ICERs), and scattered on the cost effectiveness plane to represent the sampling uncertainty around means. Statistics of incremental net monetary benefit (INMB) represent the monetary value to the NHS of individualised screening intervals per patient when the willingness-to-pay threshold is known. By rearranging the decision rule, where a treatment is cost effective if the ICER is less than the threshold, a therapy should be adopted if the INMB>0. Initially we used a lower bound threshold of £20,000 of the cost effectiveness of interventions advised by the National Institute for Health and Care Excellence,[22] as if individualised screening reported a positive INMB at £20,000, any movement toward the upper threshold of £30,000 will correspond to an increase in size and not sign. CEACs present to policymakers the likelihood of making the wrong as well as right decision; where we derived individual INMB, varied across a range of thresholds, for each bootstrapped iteration of the multivariate distributions of incremental QALYs and costs. The resulting plot presents the proportion (probability) cost effective of individualised screening by willingness-to-pay thresholds for one QALY (EQ-5D/HUI3).

Within trial subgroup analyses were not conducted due to our concerns surrounding low sample sizes,[23] and that subgroupings of interest (risk category) were inherently assigned by the risk engine and so not observed in the control arm. We are developing a risk-based model in order to investigate further.

ESM RESULTS

Secondary safety outcomes

We did not detect a clinically significant worsening of diabetes control comparing HbA1c levels across the trial arms or between the individualised arm groups (post hoc analysis). Changes in median HbA1c (mmol/mol) from to 24 months were: control 1, individualised 2 (high 2, medium1, low 2). There was no difference in the proportions of participants

in each group who had a significant (≥11mmol/mol) increase in HbA1c during the trial: control 15.2%, individualised 14.6%; high 13.4%, medium 15.0%, low 14.7%.

We did not detect differences in log minimal angle of resolution (logMAR) visual acuity (VA) (p=0.64) or in rates of visual impairment (VI) in the better eye at the last attended visit between the two arms (secondary safety outcome, ESM Table 8). Findings for worse eye VA and VI ≥ +0.50 were similar.

Health economics

At a two year time horizon we report a small number of negative QALYs. This is a drawback of health state utility tariffs utilising a scale of dead to perfect health,[24] where a select few participants reported health state utilities worse than death (<0) at each time point, producing negative QALYs. This seemed feasible given the elderly demographics of trial participants and the likelihood of multi-morbidity, and was uniformly balanced between trial arms.

As expected, all ICER point estimates fell in the south east and south west quadrants signifying the dominance of individualised screening in cost savings. Mean differences in QALYs clustered around the zero threshold across all instruments. Estimates of joint distributions following multiple imputation supported no difference or dominance in QALYs and cost savings (screening, societal). While reducing screening frequency may well improve patient quality of life, we doubt our applied instruments are sensitive enough to pick up this effect. Similarly we consider it unlikely that our time horizon is long enough to observe the benefits to those high risk patients who were confirmed STDR positive and as such received treatment sooner than they would have under annual screening. The dominance in quality of life (QoL) judged by the EQ-5D and HUI3 may be a remnant of the poor suitability of models of multiple imputation in predicting small marginal effects when missingness is high.[13]

ESM DISCUSSION – HEALTH ECONOMICS

Unlike traditional cost effectiveness analysis, individualised screening was not strictly expected to provide an increase/decrease in QALYs for an increase/decrease in cost, where the stated ICER is typically interpreted as the cost to the NHS of producing one QALY. Considering the slow progression of the development of diabetic retinopathy, and that frequency of screening appointments would plausibly have little effect on participants' health state utilities when holding safety standards constant, the observation of equivalence (or rather no skew towards QALY loss/patient across a range of sensitivity adjustments) is both expected and encouraging in viewing individualised screening as a cost minimiser. This is supported by the agreement between our multiply imputed and observed complete case utility data, and secondary safety outcomes of the ISDR trial.

This study well characterised the costs of NHS screening and treatment adherence of both standard and individualised practice that would affect screening populations such as in Liverpool. While intended to reduce patient burden, it would have been useful to collect utility data for the entire cohort. This within trial economic evaluation operated at a two year time horizon, and the effects of screening intervals on quality of life appear to be close to negligible, where our between-arm utilities and incremental QALYs demonstrated near-equivalence. A longer time horizon would be necessary to capture the possible effects on participant QoL and include blind years averted. Similarly we may not capture lifetime costing differentials (our assumptions tended to the conservative and as such may underestimate cost savings), therefore future research which fully capture diabetic retinopathy attributable treatments is advisable. Our findings provide evidence towards the characterisation of utility and costing distributions for the synthesis of evidence in health technology assessment (HTA). A risk-based model is in development to extrapolate across a lifetime horizon.

Supplementary references

1. Machin D, Campbell M, Fayers P, Pinol A. Sample Size Tables for Clinical Studies. Blackwell Science, 3rd edition, Oxford. 1997
2. Curtis L, Burns A. Unit Costs of Health and Social Care 2017. Personal Social Services Research Unit, University of Kent, Canterbury; 2017. https://doi.org/10.22024/UniKent/01.02/65559.
3. Office for National Statistics. Dataset: Consumer price inflation time series. 2019; https://www.ons.gov.uk/economy/inflationandpriceindices/datasets/consumerpriceindices
4. Department of Health and Social Care. NSRC4 NHS Trusts and PCTs Combined Reference Cost Schedules 2010-11. 2011; https://www.gov.uk/government/ publications/2010-11-reference-costs-publication
5. Little RJA, Rubin DB. Statistical Analysis with Missing Data. 2002; 2nd edition, New York: John Wiley.
6. Steyerberg EW, van Veen M. Letter: Imputation is beneficial for handling missing data in predictive models. J Clin Epidemiol. 2007;60:979.

7. National Institute for Health and Care Excellence. Position statement on use of the EQ-5D-5L value set for England (updated October 2019). 2019; https://www.nice.org.uk/about/what-we-do/our-programmes/nice-guidance/technology-appraisal-guidance/eq-5d-5l
8. Drummond M, Sculpher M, Claxton K, Stoddart G, Torrance G. Methods for the economic evaluation of health care programmes. 2015; 4th ed. Oxford University Press, Oxford.
9. Manca A, Hawkins N, Sculpher MJ. Estimating mean QALYs in trial-based cost-effectiveness analysis: the importance of controlling for baseline utility. Health Economics, 2005; 14(5), 487-496.
10. Gabrio A, Hunter R, Mason A, Baio G. Pitfalls of adjusting for mean baseline utilities/costs in trial-based cost-effectiveness analysis with missing data. 2018; arXiv:1805.07149.
11. White IR, Royston P, Wood AM. Multiple imputation using chained equations: issues and guidance for practice. Statistics in Medicine 2011; 30, 37.
12. Royston P, White IR. Multiple imputation by chained equations (MICE): implementation in Stata. J Stat Softw 2011; 45(4), 1-20.
13. Graham JW, Olchowski AE, Gilreath TD. How many imputations are really needed? Some practical clarifications of multiple imputation theory. Prev Sci 2007; 8: 206-213.
14. Stratton IM, Aldington SJ, Taylor DJ, Adler AI, Scanlon PH. A simple risk stratification for time to development of sight-threatening diabetic retinopathy. Diabetes Care. 2013 Mar;36(3):580-5. doi: 10.2337/dc12-0625.
15. MacNeil Vroomen J, Eekhout I, Dijkgraaf MG, et al. Multiple imputation strategies for zero-inflated cost data in economic evaluations: which method works best?. Eur J Health Econ 17, 939–950 (2016). https://doi.org/10.1007/s10198-015-0734-5
16. White IR., Daniel RM, Royston P. Avoiding bias due to perfect prediction in multiple imputation of incomplete categorical data. 2010; Computational Statistics & Data Analysis 54: 2267–2275.
17. Glick A, Doshi JA, Sonnad SS, Polsky D. Economic evaluation in clinical trials. 2015; 2nd ed. Oxford university press, Oxford.
18. Davidson, R; MacKinnon, JG. Estimation and inference in econometrics. 1993; Oxford University Press. ISBN 978-0-19-506011-9.
19. Rubin, DB. Multiple imputation after 18+ years. 1996; Journal of the American Statistical Association 91: 473–489.
20. Briggs A, Wonderling D, Mooney, C. Pulling cost-effectiveness analysis up by its bootstraps: a non-parametric approach to confidence interval estimation. Health Economics 1997; 6:327–340.
21. Schomaker M, Heumann C. Bootstrap inference when using multiple imputation.Statistics in Medicine. 2018; 37:2252–2266.https://doi.org/10.1002/sim.7654
22. National Institute for Health and Care Excellence. Guide to the methods of technology appraisal 2013. 2013; NICE article [PMG9]. https://www.nice.org.uk/article/pmg9/chapter/Foreword
23. Brookes ST, Whitley E, Peters TJ, Mulheran PA, Egger M, Davey Smith G. Subgroup analyses in randomised controlled trials: quantifying the risks of false-positives and false-negatives. Health Technol Assess 2001;5(33).
24. Furlong W, Feeny D, Torrance G, et al. Multiplicative Multi-Attribute Utility Function for the Health Utilities Index Mark 3 (HUI3) System: A Technical Report. Centre for Health Economics and Policy Analysis (CHEPA), McMaster University, Hamilton, Canada; 1998.

ESM Table 1. Reasons for consent not being provided

Status	Total	%
**Consent sought**	8314	100.0
**Consent provided**	4811	57.9
**Consent not provided**	3503	42.1
Does not want to take part in research	600	7.2
Does not want to be randomised	247	3.0
Does not wish to be assigned a 6 month interval	165	2.0
Does not wish to be assigned a 24 month interval	246	3.0
Patient lacking capacity	127	1.5
Already enrolled in one or more studies	18	0.2
Interpreter left due to time limitations	1	<0.1

Patient wishes to continue with annual screening	1167	14.0
No reason provided	319	3.8
Other	613	7.4

ESM Table 2. Participant baseline characteristics by arm and screening interval allocation in 4534 participants in the ITT dataset

Baseline characteristic	Arm		Baseline risk group for individualised arm			Overall total
	Control (12m)	Individualised	High	Medium	Low	
**n***	2269	2265	198	211	1856	4534
**Gender, n (%)**						
Male	1358 (59.9)	1375 (60.7)	124 (62.6)	135 (64.0)	1116 (60.1)	2733 (60.3)
Female	911 (40.1)	890 (39.3)	74 (37.4)	76 (36.0)	740 (39.9)	1801 (39.7)
**Ethnicity, n (%)**						
White	2140 (94.3)	2151 (95.0)	181 (91.4)	204 (96.7)	1766 (95.2)	4291 (94.6)
Asian	48 (2.1)	30 (1.3)	2 (1.0)	3 (1.4)	25 (1.3)	78 (1.7)
Black	40 (1.8)	43 (1.9)	6 (3.0)	3 (1.4)	34 (1.8)	83 (1.8)
Chinese	7 (0.3)	6 (0.3)	1 (0.5)	1 (0.5)	4 (0.2)	13 (0.3)
Other	25 (1.1)	29 (1.3)	8 (4.0)	0 (0.0)	21 (1.1)	54 (1.2)
Unknown	9 (0.4)	6 (0.3)	0 (0.0)	0 (0.0)	6 (0.3)	15 (0.3)
**Smoking status, n (%)**						
Smoker	419 (18.5)	371 (16.4)	26 (13.1)	39 (18.5)	306 (16.5)	790 (17.4)
Ex-smoker	877 (38.7)	909 (40.1)	69 (34.8)	76 (36.0)	764 (41.2)	1786 (39.4)
Non-smoker	965 (42.5)	981 (43.3)	103 (52.0)	96 (45.5)	782 (42.1)	1946 (42.9)
Unknown	8 (0.4)	4 (0.2)	0 (0.0)	0 (0.0)	4 (0.2)	12 (0.3)
**Diabetes type, n (%)**						
Type 1	80 (3.5)	103 (4.5)	39 (19.7)	14 (6.6)	50 (2.7)	183 (4.0)
Type 2	2024 (89.2)	1988 (87.8)	140 (70.7)	180 (85.3)	1668 (89.9)	4012 (88.5)
Unknown	165 (7.3)	174 (7.7)	19 (9.6)	17 (8.1)	138 (7.4)	339 (7.5)
**Age (years)**						
Observed, n	2269	2265	198	211	1856	4534
Median (IQR)	63.3 (55.0-71.0)	62.8 (54.9-70.3)	58.3 (49.6-66.2)	60.9 (53.4-69.8)	63.7 (56.0-70.8)	63.1 (54.9-70.7)
Range	14.1-100.7	15.4-95.7	17.5-86.8	15.4-86.8	16.8-95.7	14.1-100.7
**Disease duration (years)**						
Observed, n	2267	2262	198	209	1855	4529

	Unknown, n	2	3	0	2	1	5
	Median (IQR)	6.9 (4.2-10.9)	7.0 (4.3-11.2)	11.1 (7.3-16.1)	9.8 (6.3-13.7)	6.4 (4.0-10.1)	7.0 (4.2-11.0)
	Range	0.6-66.4	1.0-44.7	1.2-44.7	1.1-37.2	1.0-39.1	0.6-66.4
**HbA1c**							
	Observed, n	2269	2263	198	211	1854	4532
	Unknown, n	0	2	0	0	2	2
**(mmol/mol)**	Median (IQR)	51 (44-61)	52 (44-63)	67 (53-84)	58 (51-67)	50 (44-60)	51 (44-62)
	Range	26-146	28-155	33-134	34-155	28-104	26-155
**(%)**	Median (IQR)	6.8 (6.2-7.7)	6.9 (6.2-7.9)	8.3 (7.0-9.8)	7.5 (6.8-8.3)	6.7 (6.2-7.6)	6.8 (6.2-8.8)
	Range	4.5-15.5	4.7-16.3	5.2-14.4	5.3-16.3	4.7-11.7	4.5-16.3
**Systolic blood pressure (mmHg)**							
	Observed, n	2268	2265	198	211	1856	4533
	Unknown, n	1	0	0	0	0	1
	Median (IQR)	130.0 (121.0-138.0)	130.0 (122.0-138.0)	130.0 (124.0-138.0)	132.0 (124.0-140.0)	130.0 (122.0-138.0)	130.0 (122.0-138.0)
	Range	84.0-213.0	90.0-204.0	93.0-175.0	95.0-204.0	90.0-200.0	84.0-213.0
**Diastolic blood pressure (mmHg)**							
	Observed, n	2208	2206	194	201	1811	4414
	Unknown, n	61	59	4	10	45	120
	Median (IQR)	76.0 (70.0-80.0)	76.0 (70.0-80.0)	77.0 (70.0-80.0)	77.0 (70.0-80.0)	75.0 (70.0-80.0)	76.0 (70.0-80.0)
	Range	46.0-140.0	46.0-130.0	54.0-105.0	57.0-130.0	46.0-110.0	46.0-140.0
**Total cholesterol (mmol/L)**							
	Observed, n	2258	2254	197	209	1848	4512
	Unknown, n	11	11	1	2	8	22
	Median (IQR)	4.0 (3.4-4.7)	4.0 (3.4-4.7)	4.0 (3.4-4.9)	4.0 (3.4-4.6)	4.0 (3.5-4.7)	4.0 (3.4-4.7)
	Range	1.4-8.1	1.8-9.7	2.0-9.0	2.2-7.6	1.8-9.7	1.4-9.7
**Retinopathy level^, n (%)**							
	R0 R0	1857 (81.8)	1830 (80.8)	1 (0.5)	44 (20.9)	1785 (96.2)	3687 (81.3)
	R1 R0	262 (11.5)	297 (13.1)	59 (29.8)	167 (79.1)	71 (3.8)	559 (12.3)
	R1 R1	146 (6.4)	137 (6.0)	137 (69.2)	0 (0.0)	0 (0.0)	283 (6.2)

*Patients randomised who have not withdrawn and requested all data to be destroyed. ^In addition there were 5 individuals with one eye who were randomised into the trial (0.1%): 4 had R0 in one eye and were randomised into the fixed arm (0.2% of those in the fixed arm) and 1 had R1 in one eye, was randomised to the individualised arm (<0.1% of those in the individualised arm) and allocated to 6m (0.5% of those in the 6m allocation).

ESM Table 3. Withdrawals of consent, premature discontinuation of intervention and lost-to-follow-up by arm prior to first follow-up visit (primary analysis; attendance)

Withdrawals, n (%)	Arm		Overall Total
	Control	Individualised	
**Number of participants (per-protocol dataset*)**	2269 (100.0)	2234 (100.0)	4503 (100.0)
**Attendance within 90-day window known**	2224 (98.0)	2097 (93.9)	4321 (96.0)
**Attendance within 90-day window unknown**	45 (2.0)	137 (6.1)	182 (4.0)
**Withdrawal of consent**	4 (0.2)	21 (0.9)	25 (0.6)
unhappy with screening interval provided, wishes to be seen more often	0 (0.0)	16 (0.7)	16 (0.4)
no longer wished to take part	2 (0.1)	4 (0.2)	6 (0.1)
did not wish to provide reason	2 (0.1)	1 (<0.1)	3 (0.1)
**Prematurely discontinued the intervention**	0 (0.0)	15 (0.7)	15 (0.3)
**Lost-to-follow-up**	41 (1.8)	101 (4.5)	142 (3.2)
moved to a non-participating GP practice	10 (0.4)	32 (1.4)	42 (0.9)
patient died	25 (1.1)	54 (2.4)	79 (1.8)
true screen positive	0 (0.0)	0 (0.0)	0 (0.0)
opted out of screening	0 (0.0)	3 (0.1)	3 (0.1)
no longer diabetic	4 (0.2)	10 (0.4)	14 (0.3)
pregnant	2 (0.1)	1 (<0.1)	3 (0.1)
other^	0 (0.0)	1 (<0.1)	1 (<0.1)

*Note that this table excludes protocol deviations (n=31).

^This patient was on an insulin pump.

ESM Table 4. Tests for equivalence in attendance rate at 1st follow-up visit and for non-inferiority in STDR detection within 24 months, based on simple imputation of the per-protocol and intention-to-treat datasets.
In the most conservative approach all missing values are set to “not attended” and “STDR detected” and in the least conservative approach they were set to “attended” and “STDR not detected”, for the primary and secondary outcomes, respectively. The equivalence margin is predefined as δ = 0.05 and the non-inferiority margin as δ = 0.015. Proportions in both analyses (i.e., of attendance and of STDR detection) are denoted as p_C and p_I for the control and individualised arms, respectively.

Outcome	Approach		Control arm			Individualised arm			Difference in proportions $D = p_l - p_c$	95% CI of D	
			n	Attended	Proportion Attended (p_C)	n	Attended	Proportion Attended (p_I)		Lower bound (LB)	Upper bound (UB)
Primary: Attendance at 1st follow-up	Per-protocol	Most Conservative	2269	1883	0.830	2234	1754	0.785	-0.045	-0.068	0.022
		Least Conservative	2269	1928	0.850	2234	1891	0.846	-0.003	-0.024	0.018
	Intention-to-treat	Most Conservative	2269	1883	0.830	2265	1798	0.794	-0.036	-0.059	0.013
		Least Conservative	2269	1928	0.850	2265	1920	0.848	-0.002	-0.023	0.019
**Outcome**	**Approach**		**n**	**STDR Detected**	**STDR Proportion (p_C)**	**n**	**STDR Detected**	**STDR Proportion (p_I)**	D	**95% CI^ of D** LB	UB
Secondary: STDR within 24m	Per-protocol	Most Conservative	2269	262	0.115	2191	263	0.120	0.005	-0.014	0.024
		Least Conservative	2269	35	0.015	2191	28	0.013	-0.003	-0.010	0.004
	Intention-to-treat	Most Conservative	2269	262	0.115	2265	241	0.106	-0.009	-0.027	0.009
		Least Conservative	2269	35	0.015	2265	32	0.014	-0.001	-0.009	0.006

^Newcombe score 95% confidence intervals.

ESM Table 5. Test for equivalence in attendance proportion over 24 months based on the per-protocol dataset (post hoc analysis)
The equivalence margin is predefined as δ = 0.05. Proportion of attendance (denoted as p_C and p_I for the control and individualised arms, respectively) was generated from a generalised linear regression model and can be interpreted as a *weighted* mean of the mean individual attendance proportion (which is calculated as the number of attended visits divided by the number of expected visits per individual).

Outcome	Approach	Control arm		Individualised arm		Difference in proportions $D = p_l - p_c$	95% CI of D	
		n	Proportion Attended (p_C)	n	Proportion Attended (p_C)		Lower bound (LB)	Upper bound (UB)
Attendance over 24 months	Per- protocol	2224	0.848	2054	0.843	-0.005	-0.026	0.017

This table excludes 74 protocol deviations that occurred within 24 months in the individualised arm, and 45 people in the control arm, as well as 137 people in the individualised arm for whom the attendance outcome is unknown since they withdrew consent, premature discontinued the intervention or were lost-to-follow-up prior to their 1st follow-up visit.

ESM Table 6. Breakdown of the direction (increase/decrease) of intervals at first switch from baseline allocation over 24m by risk group in the individualised arm

Risk group	n^	Change in interval at 1st switch from baseline allocation	At least one change in allocation (n)	Percentage of individuals with at least one change in allocation
High	160	Change to 12m or 24m	48	30.0
Medium	200	Change to 6m	34	17.0
		Change to 24m	84	42.0
Low	1694	Change to 6m or 12m	142	8.4

^Number of individuals with a recorded attendance over 24m outcome

ESM Table 7. Withdrawals of consent, premature discontinuation of intervention and lost-to-follow-up by arm within 24 months (secondary analysis; STDR detection)

Withdrawals, n (%)	Arm		Overall Total
	Control	Individualised	
**Number of participant (per protocol dataset)***	2269 (100.0)	2191 (100.0)	4460 (100.0)
**STDR within 24m known**	2042 (90.0)	1956 (89.3)	3998 (89.6)
**STDR within 24m unknown**	227 (10.0)	235 (10.7)	462 (10.4)
**Withdrawal of consent**	22 (1.0)	35 (1.6)	57 (1.3)
unhappy with screening interval provided, wishes to be seen less often	0 (0.0)	1 (<0.1)	1 (<0.1)
unhappy with screening interval provided, wishes to be seen more often	0 (0.0)	26 (1.2)	26 (0.6)
burden of additional data collection	3 (0.1)	0 (0.0)	3 (0.1)
no longer wished to take part	6 (0.3)	4 (0.2)	10 (0.2)
did not wish to provide reason	13 (0.6)	4 (0.2)	17 (0.4)
**Prematurely discontinued the intervention**	0 (0.0)	40 (1.8)	40 (0.9)
**Lost-to-follow-up**	191 (8.4)	155 (7.1)	346 (7.8)
moved to a non-participating GP practice	40 (1.8)	38 (1.7)	78 (1.7)
patient died	67 (3.0)	65 (3.0)	132 (3.0)
true screen positive**	61 (2.7)	28 (1.3)	89 (2.0)
retained in hospital eye service – not STDR	4 (0.2)	4 (0.2)	8 (0.2)
opted out of screening	3 (0.1)	4 (0.2)	7 (0.2)
no longer diabetic	9 (0.4)	10 (0.5)	19 (0.4)

	pregnant	4 (0.2)	2 (0.1)	6 (0.1)
	Other^	3 (0.1)	4 (0.2)	7 (0.2)
**Screen positive but no biomicroscopy recorded**		14 (0.6)	5 (0.2)	19 (0.4)

*Note that this table excludes protocol deviations (n=74).
**Does not include STDR at follow-up as this is the outcome of interest.
^ Other reasons included: participant went into the digital surveillance pathway (n= 2 in each arm), was false positive but did not come back into the trial (n=1 in each arm) and was an insulin pump patient (n=1 in the individualised arm).

ESM Table 8. Descriptive table summarising secondary outcomes by arm

**Secondary outcomes**		**Arm**		**Overall**
		**Control**	**Individualised**	
**n**		2269	2191	4460
**STDR within 24m, n (%; 95% CI^)**				
	STDR	35 (1.5; 1.1-2.1)	28 (1.3; 0.9-1.8)	63 (1.4; 1.1-1.8)
	Not STDR	2007 (88.5; 87.1-89.7)	1928 (88.0; 86.6-89.3)	3935 (88.2; 87.3-89.1)
	Unknown	227 (10.0; 8.8-11.3)	235 (10.7; 9.5-12.1)	462 (10.4; 9.5-11.3)
**Attendance over 24m**				
	In trial for at least one follow-up, n	2224	2054	4278
	Did not reach first follow-up in trial, n	45	137	182
	Mean of mean individual attendance, % (95% CI)	84.8 (83.4-86.3)	83.6 (82.1-85.2)	84.3 (83.2-85.3)
**Retinopathy level at last attended screening appointment within 24m, n (%; 95% CI^)**				
	R0/R0	1509 (66.5; 64.5-68.4)	1343 (61.3; 59.2-63.3)	2852 (63.9; 62.5-65.3)
	R1/R0	239 (10.5; 9.3-11.9)	177 (8.1; 7.0-9.3)	416 (9.3; 8.5-10.2)
	R1/R1	156 (6.9; 5.9-8.0)	138 (6.3; 5.4-7.4)	294 (6.6; 5.9-7.4)
	R2/R1	8 (0.4; 0.2-0.7)	4 (0.2; 0.1-0.5)	12 (0.3; 0.2-0.5)
	R2/R2	4 (0.2; 0.1-0.5)	2 (0.1; 0.0-0.3)	6 (0.1; 0.1-0.3)
	Unassessable/R0	63 (2.8; 2.2-3.5)	23 (1.0; 0.7-1.6)	86 (1.9; 1.6-2.4)
	Unassessable/R1	10 (0.4; 0.2-0.8)	8 (0.4; 0.2-0.7)	18 (0.4; 0.3-0.6)
	Unassessable/R2	1 (<0.1; 0.0-0.3)	0 (0.0; NA)	1 (<0.1; 0.0-0.1)
	Unassessable/Unassessable	11 (0.5; 0.3-0.9)	10 (0.5; 0.3-0.8)	21 (0.5; 0.3-0.7)
	Unknown	268 (11.8; 10.6-13.2)	486 (22.2; 20.5-24.0)	754 (16.9; 15.8-18.0)
**Maculopathy level at last attended screening appointment within 24m, n (%; 95% CI^)**				

	M0/M0	1877 (82.7; 81.1-84.2)	1633 (74.5; 72.7-76.3)	3510 (78.7; 77.5-79.9)
	M1/M0	35 (1.5; 1.1-2.1)	29 (1.3; 0.9-1.9)	64 (1.4; 1.1-1.8)
	M1/M1	3 (0.1; 0.0-0.4)	2 (0.1; 0.0-0.3)	5 (0.1; 0.1-0.3)
	Unassessable/M0	71 (3.1; 2.5-3.9)	30 (1.4; 1.0-2.0)	101 (2.3; 1.9-2.7)
	Unassessable/M1	4 (0.2; 0.1-0.5)	1 (<0.1; 0.0-0.3)	5 (0.1; 0.1-0.3)
	Unassessable/Unassessable	11 (0.5; 0.3-0.9)	10 (0.5; 0.3-0.8)	21 (0.5; 0.3-0.7)
	Unknown	268 (11.8; 10.6-13.2)	486 (22.2; 20.5-24.0)	754 (16.9; 15.8-18.0)
**Screen positives within 24m, n (%; 95% CI^)**				
	Screen Positive	160 (7.1; 6.1-8.2)	105 (4.8; 4.0-5.8)	265 (5.9; 5.3-6.7)
	Screen Negative	1852 (81.6; 80.0-83.2)	1611 (73.5; 71.6-75.3)	3463 (77.6; 76.4-78.8)
	Unknown	257 (11.3; 10.1-12.7)	475 (21.7; 20.0-23.5)	732 (16.4; 15.4-17.5)
**False positives within 24m, n (%; 95% CI^)**				
	Screen Positive, n	160	105	265
	False Positive	18 (11.3; 7.2-17.1)	14 (13.3; 8.1-21.1)	32 (12.1; 8.7-16.6)
	True Positive	125 (78.1; 71.1-83.8)	81 (77.1; 68.2-84.1)	206 (77.7; 72.4-82.3)
	Condition Improved	3 (1.9; 0.6-5.4)	5 (4.8; 2.1-10.7)	8 (3.0; 1.5-5.8)
	Unknown	14 (8.8; 5.3-14.2)	5 (4.8; 2.1-10.7)	19 (7.2; 4.6-10.9)
**Visual acuity in better eye (logMAR)**				
	Observed, n	2000	1706	3706
	Unknown, n	269	485	754
	Median (IQR)	+0.02 (+0.00, +0.10)	+0.02 (+0.00, +0.10)	+0.02 (+0.00, +0.10)
	Range	[-0.30, +2.00]	[-0.30, +0.72]	[-0.30, +2.00]
**Visual acuity in worse eye (logMAR)**				
	Observed, n	2000	1706	3706
	Unknown, n	269	485	754
	Median (IQR)	+0.12 (+0.02, +0.18)	+0.12 (+0.02, +0.18)	+0.12 (+0.02, +0.18)
	Range	[-0.20, +3.00]	[-0.20, +3.00]	[-0.20, +3.00]
**Visual impairment (VA ≥ +0.30 in better eye), n (%; 95% CI^)**				
	Visually Impaired	69 (3.0; 2.4-3.8)	56 (2.6; 2.0-3.3)	125 (2.8; 2.4-3.3)
	Not Visually Impaired	1931 (85.1; 83.6-86.5)	1650 (75.3; 73.5-77.1)	3581 (80.3; 79.1-81.4)
	Unknown	269 (11.9; 10.6-13.3)	485 (22.1; 20.5-23.9)	754 (16.9; 15.8-18.0)
**Visual impairment (VA ≥ +0.50 in better eye), n (%; 95% CI^)**				

Visually Impaired	16 (0.7; 0.4-1.1)	11 (0.5; 0.3-0.9)	27 (0.6; 0.4-0.9)
Not Visually Impaired	1984 (87.4; 86.0-88.7)	1695 (77.4; 75.6-79.1)	3679 (82.5; 81.4-83.6)
Unknown	269 (11.9; 10.6-13.3)	485 (22.1; 20.5-23.9)	754 (16.9; 15.8-18.0)
**Number of screening appointments / Number of non-attended screening appointments, n (%; 95% CI)**			
Total number of screening appointments, n	5034	3074	8108
Attended, n (%; 95% CI)	3536 (70.2; 69.0-71.5)	2008 (65.3; 63.6-67.0)	5544 (68.4; 67.4-69.4)
Did Not Attend, n (%; 95% CI)	1498 (29.8; 28.5-31.0)	1066 (34.7; 33.0-36.4)	2564 (31.6; 30.6-32.6)

^Wilson score confidence intervals.

ESM Table 9. Numbers of attended follow-up appointments and screen positive events within 24 months (+90 day window) by arm using the per-protocol dataset at 24 months

Screen Positive Status	Arm		Risk Group within Individualised Arm			Total
	Control	Individualised	High	Medium	Low	
Total number of individuals, n	2269	2191	162	203	1826	4460
Number of attended follow-up appointments, n (%)	3536 (100.0)	2008 (100.0)	317 (100.0)	249 (100.0)	1442 (100.0)	5544 (100.0)
Screen positive, n (%)	160 (4.5)	102 (5.1)	34 (10.7)	15 (6.0)	53 (3.7)	262 (4.7)
Screen positive for DR, n (%)	54 (1.5)	45 (2.2)	29 (9.1)	9 (3.6)	7 (0.5)	99 (1.8)
Screen positive for unassessable images, n (%)	79 (2.2)	39 (1.9)	3 (0.9)	5 (2.0)	31 (2.1)	118 (2.1)
Screen positive for other eye disease requiring HES, n (%)	27 (0.8)	18 (0.9)	2 (0.6)	1 (0.4)	15 (1.0)	45 (0.8)
Screen negative, n (%)	3371 (95.3)	1905 (94.9)	283 (89.3)	234 (94.0)	1388 (96.3)	5276 (95.2)
Unknown status, n (%)	5 (0.1)	1 (<0.1)	0 (0.0)	0 (0.0)	1 (0.1)	6 (0.1)

ESM Table 10. Micro-costing of the Liverpool Diabetes Eye Screening Programme and estimated costs of the Risk Calculation Engine

Variable		Top-down annual total	Per attendance (n=16736)	Per non-attendance (n=6179)
**Programme costs**				
	Staff (including oncosts)	£242,715	£10.59	£10.59
	Stationary	£3,056	£0.13	£0.13
	IT	£22,988	£1.00	£1.00
	**Total**	£268,759	£11.73	£11.73
**Photography**				
	Staff	£157,528	£9.08	£0.91
	Cameras and equipment	£11,777	£0.68	£0.07
	Medical consumables	£4,229	£0.25	£0.00
	**Total**	£173,534	£10.00	£1.00
**Grading (P3-P6)**				
	Staff	£117,234	£7.00	£0.00
**Total NHS cost**		£559,528	**£28.73**	**£12.73**
**Societal costs associated with screening attendance**				
	Patient-borne costs	-	£2.64	-
	Productivity loss	-	£6.36	-
**Total societal cost**		-	**£9.00**	-

**Risk calculation engine (RCE) costs**		**Annual total**	**Per patient**
	Database administrator	£34,000	£1.54
	CCG administrator (20% FTE)	£6,000	£0.27
	**RCE total**	£40,000	**£1.81**

Not all values sum perfectly due to rounding. Estimated costs to run and maintain a data warehouse and risk engine based on Liverpool eligible population of 22,099. Excludes start-up costs.

ESM Table 11. Control arm summary statistics of intention-to-treat health economic data at 24 months

Variable	**Control arm** (*n*=2269)					
	n	Mean	SD/95% CI	Min.	Median	Max.
**Baseline**						
EQ-5D	400	0.70	0.30	-0.27	0.77	0.99
EQ-VAS	403	0.74	0.22	-0.90	0.80	1.00
HUI3	350	0.70	0.30	-0.21	0.79	1.00
**6 months**						
EQ-5D	9	0.00	0.00	0.00	0.00	0.00
EQ-VAS	9	0.00	0.00	0.00	0.00	0.00
HUI3	11	0.00	0.00	0.00	0.00	0.00
**12 months**						
EQ-5D	271	0.66	0.30	-0.29	0.73	0.99
EQ-VAS	274	0.71	0.25	0.00	0.75	1.00
HUI3	253	0.65	0.35	-0.22	0.75	1.00
**18 months**						
EQ-5D	57	0.44	0.41	-0.23	0.55	0.98
EQ-VAS	60	0.51	0.38	0.00	0.65	1.00
HUI3	60	0.38	0.43	-0.27	0.27	1.00
**24 months**						
EQ-5D	284	0.64	0.34	-0.23	0.73	0.99
EQ-VAS	288	0.69	0.27	0.00	0.75	1.00
HUI3	253	0.63	0.36	-0.27	0.75	1.00
**Total QALYs**						
**EQ-5D**						
Unadjusted	278	1.33	0.60	-0.40	1.49	1.96
Baseline adjusted	278	1.34	(1.30 to 1.37)	-	-	-
**EQ-VAS**						
Unadjusted	281	1.42	0.45	0.09	1.53	2.00
Baseline adjusted	281	1.40	(1.36 to 4.43)	-	-	-
**HUI3**						
Unadjusted	216	1.36	0.62	-0.37	1.55	2.00
Baseline adjusted	216	1.36	(1.32 to 1.41)	-	-	-
**Total costs (£)**						
Non-attendance	2194	9.24	(8.61 to 9.88)	0.00	0.00	76.38
Screening	2194	85.54	(84.86 to 86.25)	28.73	86.19	137.11
Societal	2194	109.44	(108.61 to 110.29)	37.73	113.19	164.11

EQ-5D = Euroqol five dimension five level instrument mapped to the three level value set, EQ-VAS = Euroqol visual analogue scale, HUI3 = Health Utilities Index Mark 3. Screening costs per patient include the costs of non-attendance. Societal costs are the combination of NHS screening costs, productivity losses, and out-of-pocket expenses. We present 95% confidence intervals in place of standard deviations for highly skewed distributions, such as costs, and for QALYs where necessitated by regression adjustment.

ESM Table 12: Individualised arm summary statistics of intention-to-treat health economic data at 24 months

Variable	**Individualised arm** (*n*=2265)					
	n	Mean	SD/95% CI	Min.	Median	Max.
**Baseline**						
EQ-5D	399	0.70	0.30	-0.20	0.77	0.99
EQ-VAS	403	0.73	0.22	0.05	0.78	1.00
HUI3	352	0.71	0.30	-0.21	0.82	1.00
**6 months**						
EQ-5D	35	0.38	0.43	-0.30	0.30	0.99
EQ-VAS	33	0.47	0.42	0.00	0.50	1.00
HUI3	35	0.38	0.43	-0.13	0.06	1.00
**12 months**						
EQ-5D	61	0.55	0.42	-0.14	0.67	0.99
EQ-VAS	60	0.60	0.35	0.00	0.70	1.00
HUI3	60	0.54	0.42	-0.15	0.72	1.00
**18 months**						
EQ-5D	39	0.48	0.45	-0.10	0.65	0.98
EQ-VAS	40	0.54	0.41	0.00	0.70	1.00
HUI3	39	0.47	0.44	0.00	0.55	1.00
**24 months**						
EQ-5D	270	0.65	0.35	-0.41	0.76	0.99
EQ-VAS	269	0.68	0.27	0.00	0.75	1.00
HUI3	226	0.63	0.37	-0.28	0.78	1.00
**Total QALYs**						
**EQ-5D**						
Unadjusted	261	1.35	0.62	-0.60	1.55	1.97
Baseline adjusted	261	1.34	(1.30 to 1.38)	-	-	-
**EQ-VAS**						
Unadjusted	267	1.38	0.48	0.05	1.55	2.00
Baseline adjusted	267	1.40	1.37 to 1.44	-	-	-
**HUI3**						
Unadjusted	192	1.35	0.66	-0.34	1.64	2.00
Baseline adjusted	192	1.35	(1.30 to 1.39)	-	-	-
**Total costs (£)**						
Non-attendance	2195	6.77	(6.20 to 7.39)	0.00	0.00	101.84
Screening	2195	68.10	(67.15 to 69.05)	32.35	61.08	207.65
Societal	2195	86.18	(84.99 to 87.31)	41.35	79.08	243.65

EQ-5D = Euroqol five dimension five level instrument mapped to the three level value set, EQ-VAS = Euroqol visual analogue scale, HUI3 = Health Utilities Index Mark 3. Screening costs per patient include the costs of non-attendance. Societal costs are the combination of NHS screening costs, productivity losses, and out-of-pocket expenses. We present 95% confidence intervals in place of standard deviations for highly skewed distributions, such as costs, and for QALYs where necessitated by regression adjustment.

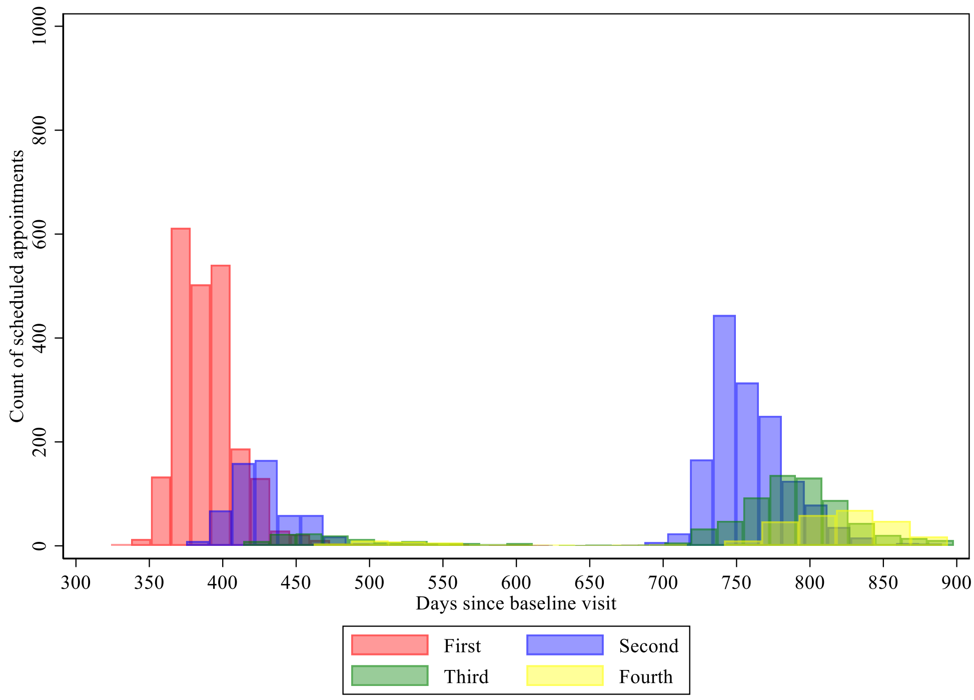

ESM Fig 1. Control arm distributions (frequency) of scheduled screening appointments

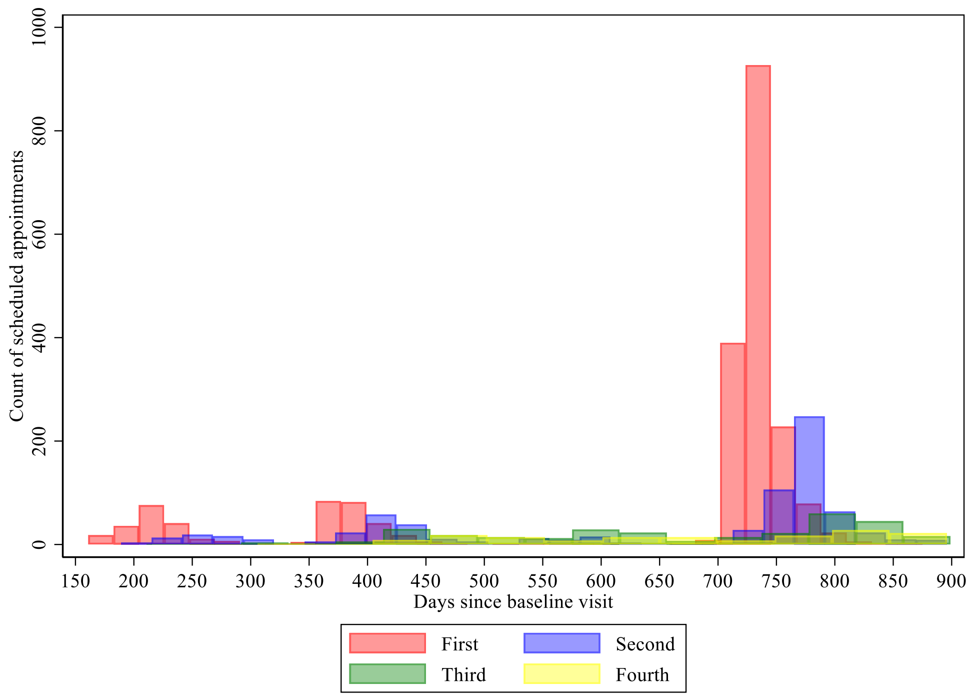

ESM Fig 2. Individualised arm distributions (frequency) of scheduled screening appointments

MEMBERS OF THE ISDR STUDY GROUP NOT QUALIFYING AS AUTHORS

ISDR investigators:
Anthony C Fisher (RCE lead), Department of Medical Physics and Clinical Engineering, Royal Liverpool University Hospital, UK
Paula Byrne (Qualitative and PPI lead), Institute of Population Health, University of Liverpool, University of Liverpool, UK
Paul Grey (NHS manager representative), Joint Director of Public Health, Liverpool PCT/Liverpool City Council, UK
Daniel Seddon (NHS manager representative), Public Health England and NHS England, Cheshire and Merseyside Screening and Immunisation, UK
Paula Williamson (Director), Liverpool Clinical Trials Research Centre, University of Liverpool, UK

ISDR Research staff:
Duncan Appelbe (Information systems manager), Liverpool Clinical Trials Research Centre, University of Liverpool, UK
Ayesh Alshukri (Computer scientist, RCE team), Department of Eye and Vision Science, University of Liverpool, UK
Antonio Eleuteri (Computer scientist, RCE team), Department of Medical Physics and Clinical Engineering, Royal Liverpool University Hospital, UK
Christopher Grierson (ISDR admin and data management teams), St Paul's Eye Unit, Royal Liverpool University Hospital, UK
Lola Howard (Trial Coordinator), Liverpool Clinical Trials Research Centre, University of Liverpool, UK
Susan U Howlin (Senior data manager), Liverpool Clinical Trials Research Centre, University of Liverpool, UK
Mehrdad Mobayen-Rahni, Department of Medical Physics and Clinical Engineering, Royal Liverpool University Hospital, UK
Andrew Ovens (Information Systems Developer), Liverpool Clinical Trials Research Centre, University of Liverpool, UK
Christopher J Sampson (Health Economist), Division of Rehabilitation, Ageing and Wellbeing, University of Nottingham and The Office of Health Economics, UK
Kate Silvera (Data manager), Liverpool Clinical Trials Research Centre, University of Liverpool, UK
David Szmyt (ISDR admin and data management teams), St Paul's Eye Unit, Royal Liverpool University Hospital, UK
Clare Thetford (Research fellow), Department of Health Services Research, University of Liverpool, UK
Pilar Vazquez-Arango (Programme manager, 2018-2020), Department of Eye and Vision Science, University of Liverpool, UK
Abigail E Williams (Trial Coordinator), Liverpool Clinical Trials Research Centre, University of Liverpool, UK

Patient and Public Involvement Group:
John Collins (Patient representative), ISDR Patient and Public Involvement Group, University of Liverpool, UK
John Kelly (Patient representative), ISDR Patient and Public Involvement Group, University of Liverpool, UK
Peter Lees (Patient representative), ISDR Patient and Public Involvement Group, University of Liverpool, UK
Sandra Lees (Patient representative), ISDR Patient and Public Involvement Group, University of Liverpool, UK
Emily Doncaster (Patient representative), ISDR Patient and Public Involvement Group, University of Liverpool, UK
Betty Williams (Patient representative), ISDR Patient and Public Involvement Group, University of Liverpool, UK

ISDR Programme Steering Committee:
Gideon Smith (Chair), Director of Public Health, Tameside Metropolitan Borough Council, UK
Naveed Younis (Independent Diabetologist), Wythenshawe Hospital, Manchester University NHS Foundation Trust, UK
Julia West (representing sponsor), Research Development & Innovation, Royal Liverpool University Hospital, UK
Alison Rowlands (Independent Ophthalmologist), Arrowe Park Hospital, Wirral University Teaching Hospital NHS Foundation Trust, UK
Catey Bunce (Independent Statistician), School of Population Health and Environmental Sciences, King's College London, UK

Helen Cooper (Independent Academic), Department of Health and Social Care, University of Chester, Chester, UK

Independent Data Safety Committees:
Chris Rogers (Chair), Bristol Heart Institute, University of Bristol, UK
Vineeth Kumar (Independent Ophthalmologist), Arrowe Park Hospital, Wirral University Teaching Hospital NHS Foundation Trust, UK
Nathalie Massat (Independent Statisitician), Wolfson Institute of Preventive Medicine, Queen Mary University of London, UK

Liverpool Diabetic Eye Screening Programme:
Ticiana Criddle (Director), Liverpool Diabetic Eye Screening Programme, Royal Liverpool University Hospital, UK
Stephanie Perrett, Liverpool Diabetic Eye Screening Programme, Royal Liverpool University Hospital, UK

Liverpool Clinical Commissioning Group:
Lisa Jones (Primary Care Systems Analyst), NHS Liverpool Clinical Commissioning Group, UK
